# Attenuation of chronic antiviral T-cell responses through constitutive COX2-dependent prostanoid synthesis by lymph node fibroblasts

**DOI:** 10.1371/journal.pbio.3000072

**Published:** 2019-07-15

**Authors:** Karin Schaeuble, Hélène Cannelle, Stéphanie Favre, Hsin-Ying Huang, Susanne G. Oberle, Daniel E. Speiser, Dietmar Zehn, Sanjiv A. Luther

**Affiliations:** 1 Center for Immunity and Infection Lausanne, Department of Biochemistry, University of Lausanne, Epalinges, Switzerland; 2 Department of Oncology, University of Lausanne and University Hospital, Epalinges, Switzerland; 3 Swiss Vaccine Research Institute, Epalinges, Switzerland; 4 Division of Immunology and Allergy, Department of Medicine, Lausanne University Hospital, Lausanne, Switzerland; 5 Division of Animal Physiology and Immunology, School of Life Sciences Weihenstephan, Technical University of Munich, Freising, Germany; National Cancer Institute, UNITED STATES

## Abstract

Lymphoid T-zone fibroblastic reticular cells (FRCs) actively promote T-cell trafficking, homeostasis, and expansion but can also attenuate excessive T-cell responses via inducible nitric oxide (NO) and constitutive prostanoid release. It remains unclear how these FRC-derived mediators dampen T-cell responses and whether this occurs in vivo. Here, we confirm that murine lymph node (LN) FRCs produce prostaglandin E_2_ (PGE_2_) in a cyclooxygenase-2 (COX2)-dependent and inflammation-independent fashion. We show that this COX2/PGE_2_ pathway is active during both strong and weak T-cell responses, in contrast to NO, which only comes into play during strong T-cell responses. During chronic infections in vivo, PGE_2_-receptor signaling in virus-specific cluster of differentiation (CD)8 cytotoxic T cells was shown by others to suppress T-cell survival and function. Using COX2^flox/flox^ mice crossed to mice expressing Cre recombinase expression under control of the CC chemokine ligand (CCL19) promoter (CCL19cre), we now identify CCL19^+^ FRC as the critical source of this COX2-dependent suppressive factor, suggesting PGE_2_-expressing FRCs within lymphoid tissues are an interesting therapeutic target to improve T-cell–mediated pathogen control during chronic infection.

## Abstract 2

Fibroblasts in secondary lymphoid organs can be active participants in adaptive immunity, often enhancing T-cell responses. This study shows how these fibroblasts dampen T-cell responses via the constitutive production of the COX2-dependent prostaglandin PGE2, including during persistent viral infection.

## Introduction

Lymph nodes (LNs) are secondary lymphoid organs (SLOs) specialized in filtering lymph fluid and initiating T- and B-cell responses to foreign antigens. Typically, antigen-specific T cells are selected to expand and differentiate into effector cells by dendritic cells (DCs) that present processed antigen in the context of major histocompatibility complex (MHC) and costimulatory signals. However, T cells may also be tolerized if self-antigens are presented in a nonimmunogenic context, typically by immature DCs. All of these processes take place within the T-cell–rich compartment of SLOs that are organized by fibroblastic reticular cells (FRCs), which are the most prevalent nonhematopoietic cell type in this zone and play active immune regulatory roles [1, 2].

Initially, FRCs have mainly been described to enhance adaptive immunity in several ways. FRCs constitutively produce the chemokines CCL19 and CCL21 responsible for attracting and retaining DCs and naïve T cells in that compartment and thereby facilitating their physical interaction [3, 4]. These encounters are further enhanced by the dense reticular network formed by FRCs, allowing DC adhesion and physical guidance for migrating T cells during their continuous search for antigen [5, 6]. FRCs play yet another important role in promoting adaptive immunity by producing the T-cell survival factor interleukin-7 (IL-7) [3].

More recently, evidence has accumulated for negative regulatory roles of FRCs in T-cell responses [1, 2]. FRCs were shown to express self-antigens in the context of MHCI [7] or to acquire antigens and MHCII molecules from neighboring DCs [8], with evidence suggesting induction of peripheral T-cell tolerance. However, FRCs can also inhibit T-cell responses as bystander cells, presumably without need of antigen presentation. Ex vivo, FRCs were demonstrated to express inducible nitric oxide synthase (iNOS) upon detection of interferon gamma (IFNγ) and tumor necrosis factor alpha (TNFα) derived from T cells shortly after priming, leading to a nitric oxide (NO)-dependent attenuation of T-cell proliferation [9–11]. This mechanism was shown to be limited to the early phase of T-cell priming, leading to a reduced expansion of T cells that were nevertheless functional. While strong in vivo evidence for a critical role of iNOS or self-antigen in FRC dampening T-cell responses is still scarce, these findings suggest that FRCs may protect tissues against damage caused by very strong and potentially pathogenic T-cell responses.

During our initial studies with FRC lines, we observed that part of the inhibitory activity was dependent on cyclooxygenase (COX) 1 and/or 2 enzymes because it could be blocked by the pharmacological inhibitor indomethacin. Interestingly, LN FRC line COX2 expression is constitutive, in contrast to inflammation-induced iNOS expression [11]. This initial finding suggested that FRCs may have multiple pathways capable of dampening T-cell responses, but the relative roles of COX versus iNOS in FRCs remained unclear, as did the precise context, mechanism, and in vivo role of COX2, specifically in FRCs.

COX enzymes are the rate-limiting enzymes in the pathway converting arachidonic acid into various prostanoids (COX-dependent lipids), namely prostaglandins and thromboxane A2. Two genes code for two COX enzymes that differ in their expression pattern but perform the same function. Typically, COX1 is constitutively expressed in several cell types to support homeostatic processes, while COX2 is transiently up-regulated during inflammatory reactions in injured tissues or infiltrating myeloid cells. Among the COX-dependent lipids, prostaglandin E_2_ (PGE_2_) has powerful proinflammatory activities responsible for many hallmarks of inflammation, including fever, pain, and swelling, acting via one of the four receptors, E Prostanoid (EP) 1–4 [12–14]. Many nonsteroidal anti-inflammatory drugs (NSAIDs), including aspirin, ibuprofen, indomethacin, and paracetamol, target this function of the two COX enzymes. They are among the most widely used drugs and have become a standard treatment for many inflammatory reactions. However, several reports have highlighted the dual function of COX-dependent prostanoids in inflammation [12–16]. During acute inflammation, PGE_2_ enhances innate immunity through local vasodilation and recruitment of neutrophils and macrophages. However, PGE_2_ is also prevalent during chronic inflammation, such as cancer and persisting, nonresolved viral infection, in which it is anti-inflammatory and suppresses type I immunity [17–19]. In addition, PGE_2_-induced effects appear to be strongly dose- and context-dependent, and T-cell suppression correlates with high PGE_2_ concentrations [14, 20]. These findings emphasize the importance of understanding how COX-dependent prostanoids regulate immune responses in vivo to improve mechanistic knowledge of the widely used COX-inhibiting drugs.

In this study, we show that LN FRCs constitutively express high levels of COX2 and its product PGE_2_, thereby dampening T-cell expansion in vitro. This property of FRCs is independent of the strength of the inflammatory stimulus, in contrast to the second negative regulator, NO. Importantly, mice lacking FRC COX2 are unable to attenuate T-cell responses during persisting viral infection. These findings suggest that the EP2/4-dependent mechanism of T-cell suppression previously observed by Kaech and colleagues during chronic lymphocytic choriomeningitis virus (LCMV) clone 13 infection [18] is mediated by COX2^+^ LN FRCs, pointing to a previously unappreciated role for FRCs as negative regulators of chronic immune responses.

## Results

### LN FRCs dampen CD8+ and CD4+ T-cell responses in vitro via constitutive COX2-dependent prostanoid production

**Fig 1:**
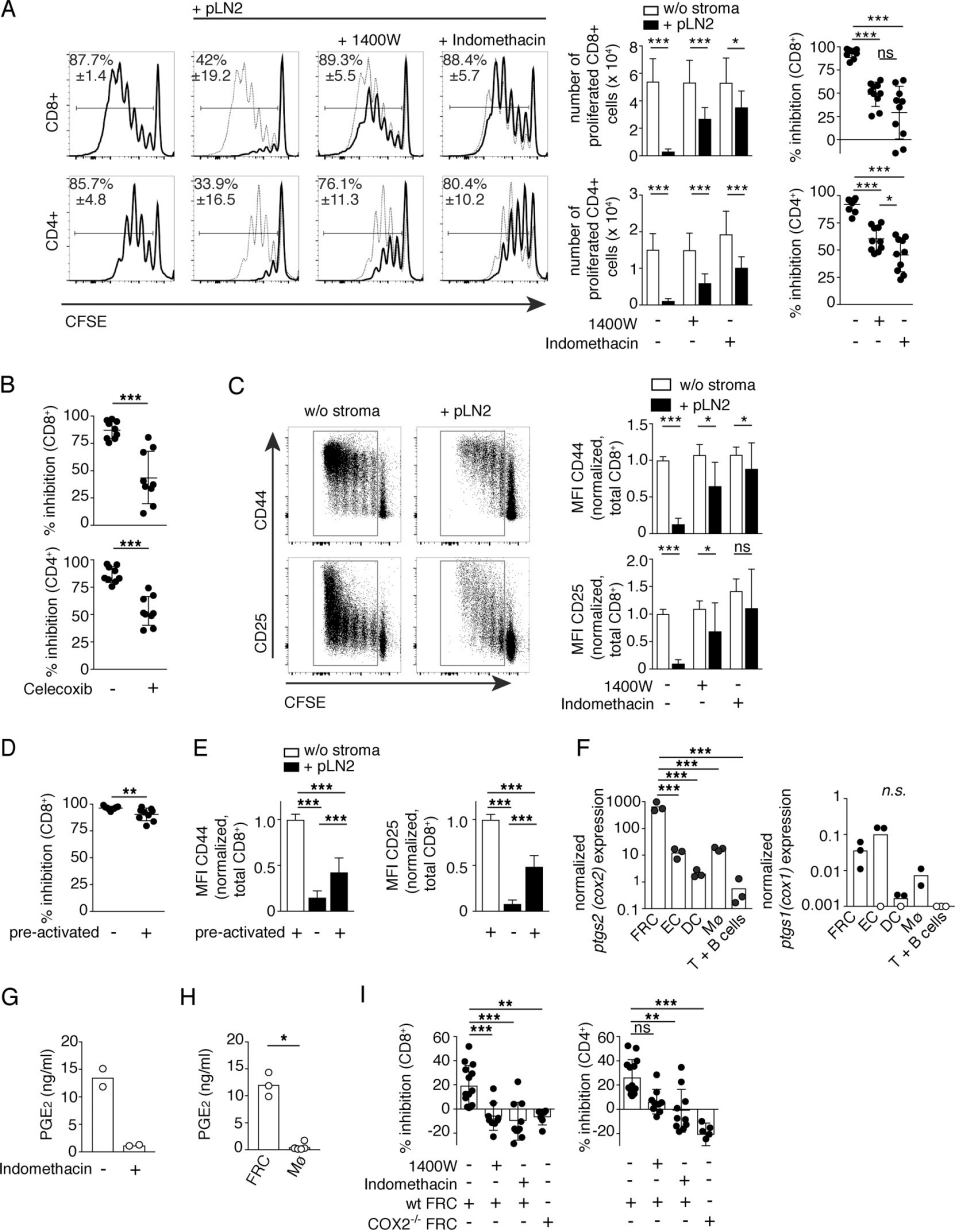
Naïve LN FRCs dampen CD8 and CD4 T-cell activation and proliferation by constitutive COX2 and PGE_2_ expression. (A–E) CFSE-labeled T cells were activated nonspecifically with αCD3/28 DynaBeads and cultured in the absence or presence of pLN2 (pLN FRC cell line) and inhibitors, followed by flow cytometric analysis. (A–C) 3 d coculture of lymphocytes ± pLN2 ± inhibitor. (A) Shown on the left side are histograms of CD8+ and CD4+ T cells depicting CFSE dilution as readout of T-cell proliferation in presence (thick line) or absence (thin line) of FRCs ± iNOS inhibitor 1400W (3 μM) or COX1/2 inhibitor indomethacin (10 μM). Bar graphs in the middle indicate the number of proliferated CD8+ and CD4+ T cells. Scatter plots on the right side represent the percentage inhibition of T-cell proliferation mediated by FRCs, calculated based on the number of T cells 3 d after coculture. (B) Coculture of lymphocytes ± pLN2 ± celecoxib (5 μM). Scatter plots depict the percentage inhibition of CD8+ and CD4+ T-cell proliferation in presence of pLN2. (C) Left side: dot plots showing CD44 or CD25 expression versus CFSE dilution among CD8+ T cells after coculture. Right side: bar graphs depicting the MFI of CD44 and CD25 on total CD8+ T cells with different data sets normalized to the untreated condition in absence of stroma (set at 1.0) to allow pooling of the data sets and better statistical analysis. Data shown in (A) and (C) represent four independent experiments (*n* = 10). (D) CFSE-labeled T cells preactivated nonspecifically for 24 h with αCD3/28 DynaBeads were cultured thereafter for 2 d ± pLN2 cells. Inhibition of CD8+ T-cell proliferation mediated by pLN2 cells is shown. (E) Bar graphs illustrating the MFI of CD44 and CD25 of total CD8+ T cells cultured as described in (D). Data were normalized to the MFI of CD8+ T cells cultured 3 days in the absence of pLN2 cells. Data shown in (B), (D), and (E) represent 3 independent experiments (*n* = 9). (F) RT-qPCR analysis for *ptgs1/2* transcript levels in the indicated sorted cell types from pLN of naïve WT mice (*n* = 2–3; each sample represents a pool of 2–3 mice). Transcript levels below the detection limit or nonspecific transcripts are indicated as white circles on the x-axis. (G, H) PGE_2_ levels in the supernatant after overnight culture as determined by ELISA. (G) pLN2 cells cultured in the presence or absence of indomethacin (10 μM). (H) FRC (CD45− CD31− pdpn+) and CD11b+ macrophages sorted from pLN of mice SC immunized 5.5 d earlier with the foreign antigen OVA in Montanide adjuvant. In the case of macrophage cultures, GM-CSF was added (*n* ≥ 3). (I) CFSE-labeled T cells were activated nonspecifically with αCD3/28 DynaBeads and cultured for 3 d ± FRCs isolated ex vivo from pLN of WT and COX2^−/−^ mice, respectively. Bar graphs depict percentage inhibition of CD8+ and CD4+ T-cell proliferation mediated by ex vivo FRCs ± indomethacin (10 μM) or 1400W (3 μM), respectively. *n* ≥ 6; pool of 2–3 independent experiments. Bar graphs and scatter dot plots show the mean ± SD. Statistics: to compare two groups (A, B, C, D, and H), unpaired *t* test and Mann–Whitney test were used. For comparison of multiple groups (A, E, F, and I), we used one-way ANOVA or Kruskal–Wallis tests with multiple comparisons. *P < 0.05, **P < 0.005, and ***P < 0.001. Data used in the generation of this figure can be found in S1 Data. CD, cluster of differentiation; CFSE, carboxyfluorescein succinimidyl ester; COX, cyclooxgyenase; d, day; FRC, fibroblastic reticular cell; GM-CSF, Granulocyte-Macrophage Colony-Stimulating Factor; iNOS, inducible nitric oxide synthase; LN, lymph node; MFI, median fluorescence intensity; Mø, macrophage; ns, not significant; OVA, ovalbumin; pdpn, podoplanin; PGE_2_, prostaglandin E_2_; pLN, peripheral LN; *ptgs*, prostaglandin-endoperoxide synthase; RT-qPCR, reverse transcription followed by a quantitative polymerase chain reaction; WT, wild type; w/o, without.

We previously described the COX-dependent T-cell expansion attenuation in a coculture system in which T cells were activated by antigen-loaded DCs in the presence of FRCs [11]. To exclude a role for DCs as a source of COX-dependent prostanoids and to study the role of COX2 in FRCs, we activated naïve murine T cells using α cluster of differentiation (CD)3/28-coated beads in the presence or absence of the FRC line called peripheral LN (pLN)2. The percentage and absolute numbers of proliferating CD8+ and CD4+ T cells were attenuated 85%–95% in presence of FRCs, and this inhibitory effect could be partially reversed by adding the iNOS inhibitor 1400W, the COX1/2-inhibitor indomethacin (Fig 1A), or the COX2-specific inhibitor celecoxib (Fig 1B). The inhibitors enhanced the CD4+ and CD8+ T-cell expansion around 3- to 5-fold, with the response reaching 35%–70% of the T-cell response observed in the absence of FRCs (Fig 1A and 1B) and with a concomitant increased expression of the activation markers CD44 and CD25 on the total T-cell population (Fig 1C; S1A and S1B Fig). Interestingly, among the proliferating T cells, there were only small differences in CD44 and CD25 expression upon addition of inhibitors (S1A and S1B Fig), suggesting FRC-expressed iNOS and COX2 reduce the number of primed T cells and have less effect on T cells once they have successfully entered the cell cycle. To test this hypothesis more directly, T cells preactivated for 24 h using αCD3/28-coated beads were cocultured with pLN2 cells and compared with naïve T cells. Even preactivated CD8+ and CD4+ T cells were potently suppressed by FRCs, as assessed by carboxyfluorescein succinimidyl ester (CFSE) dilution and up-regulation of activation markers, although to a slightly decreased extent relative to naïve T cells (Fig 1D and 1E; S1C and S1D Fig).

### FRC-derived PGE_2_ is responsible for inhibiting T-cell expansion in an EP2/4-receptor–dependent fashion

To investigate the conditions under which the iNOS and COX pathways become active in LN FRCs, we initially analyzed the transcript levels of these enzymes in pLN2 cells as well as ex vivo LN FRCs. Consistent with previous data [9–11], the gene coding iNOS, *Nos2*, was not detectable in naïve pLN2 but was strongly induced 7 h after stimulation with IFNγ/TNFα or lipopolysaccharide (LPS), while prostaglandin-endoperoxide synthase 2 (*ptgs2*) (COX2) was highly expressed in unstimulated pLN2 with only a small increase upon cytokine stimulation (S1E Fig). Similarly, *ptgs1* (COX1) transcripts were constitutively expressed in pLN2 but at 10-fold lower levels than *ptgs2* (S1E Fig), suggesting COX2 is the isoform preferentially expressed in pLN2 cells and responsible for the attenuating effect observed on T-cell expansion. To test whether this holds true in vivo, pLNs and spleens from adult wild-type (WT) mice were passed through a mesh to enrich either for lymphocytes or “nonsoluble” stromal cells [4]. Transcripts for both COX isoforms were enriched 5- to 50-fold in the stromal cell fraction of pLNs, with less-marked differences observed for the splenic fractions (S1F Fig). To identify the stromal cell types expressing *ptgs1/2* in naïve pLNs, various cell populations from naïve pLNs were purified and analyzed. While *ptgs1* transcripts were found at similar levels in FRCs and endothelial cells, *ptgs2* transcripts were expressed at 100-fold higher levels in FRCs relative to endothelial, myeloid, or lymphoid cells (Fig 1F). Notably, transcript levels of *ptgs2* were approximately 1,000-fold higher in FRCs than those of *ptgs1*, indicating COX2 is the principle isoform expressed by naïve pLN FRCs.

COX enzymes are critical for the production of lipid intermediates that are further metabolized to produce the different effector molecules of the prostaglandin and thromboxane family. Previous studies have shown that high COX2 expression often correlates with high levels of PGE_2_, with this prostanoid being known to inhibit T-cell activation and proliferation in various settings [12–16]. Because PGE_2_ production depends on prostaglandin E synthases (ptges), we assessed the transcript levels of all three isoforms (*ptges1–3*). Both in naïve pLNs and spleens, all three isoforms were expressed, with *ptges1* and *ptges3* being preferentially expressed in the stromal cell fraction (S1G Fig). Consistent with constitutive activity of this pathway in FRC, overnight culture of the pLN2 FRC cell line led to the accumulation of 10–15 ng/ml PGE_2_ in the supernatant, which was abolished in the presence of indomethacin (Fig 1G). In addition, pLN FRCs were isolated and sorted from mice immunized 5 days (d) earlier with the foreign antigen ovalbumin (OVA) in Montanide adjuvant, and these FRCs produced high concentrations of PGE_2_ as well, about 10-fold more than macrophages isolated from the same LN (Fig 1H). These findings point to FRCs being a rich constitutive PGE_2_ source in naïve and activated pLNs. Indeed, FRCs isolated ex vivo from pLNs of naïve WT mice also inhibited CD8+ and CD4+ T-cell proliferation, to a lesser extent than the FRC line but still in an iNOS- and Cox1/2-dependent manner, as shown using pharmacological inhibitors or FRCs isolated from Cox2^−/−^ FRCs (Fig 1I).

**Fig 2:**
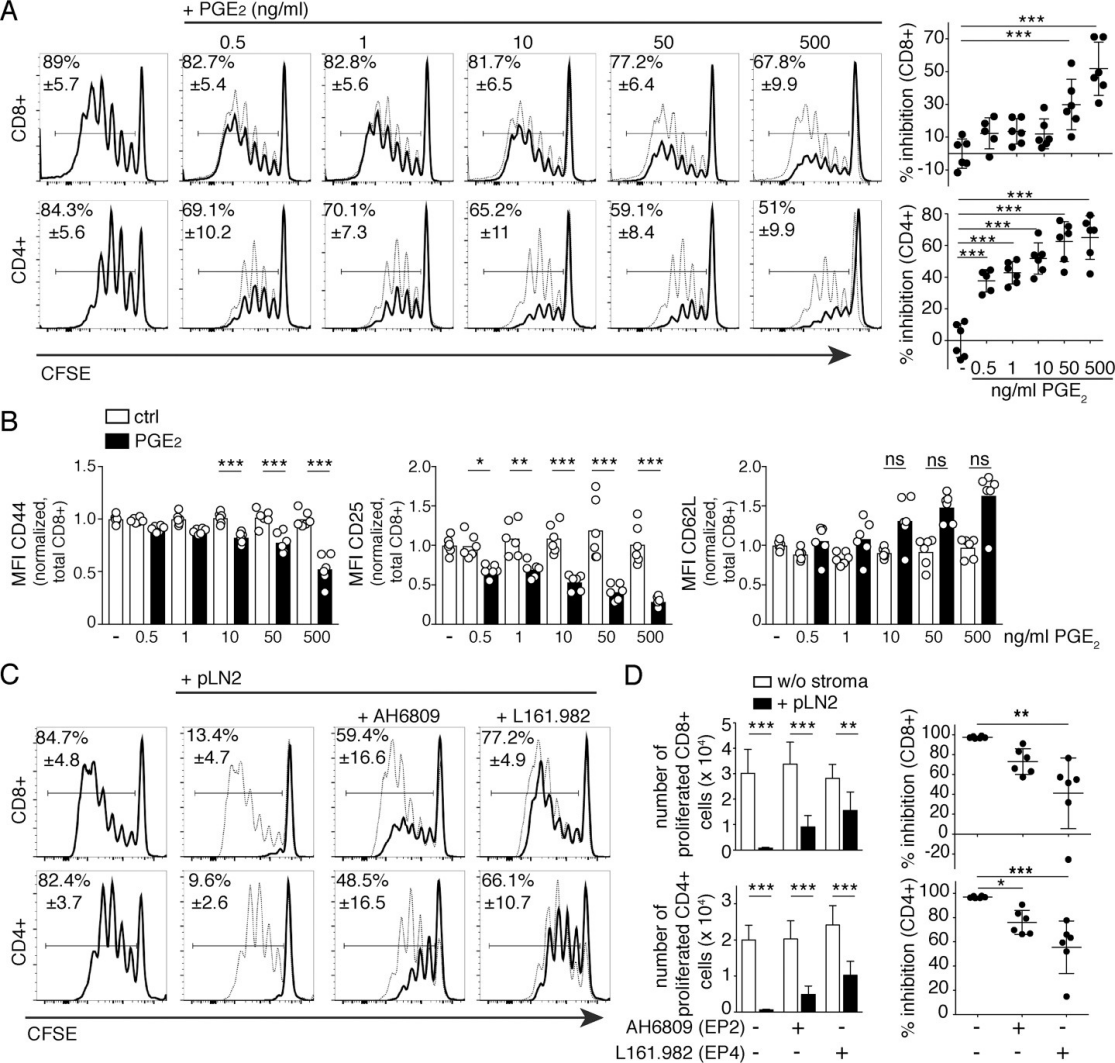
LN FRCs constitutively produce the COX-dependent mediator PGE_2_, which enables inhibition of T-cell activation and proliferation mainly via EP4 receptor signaling. Flow cytometric analysis of CFSE dilution in CD8+ and CD4+ T cells that were activated with αCD3/28 DynaBeads and cultured for 3 d. (A) Left side: CFSE dilution profiles of T cells in presence of the indicated concentrations of PGE_2_ (black line) or solvent control (ethanol; thin line), with, on the right side, scatter plots showing the percentage inhibition of T-cell proliferation. (B) Expression level (MFI) of CD44, CD25, and CD62L on total CD8+ T cells in presence of PGE_2_. (C) CFSE profile of T cells cultured in presence (thick line) or absence (thin line) of pLN2 cells and the EP2 antagonist AH6809 (5 μM) or the EP4 antagonist L161.982 (5 μM). (D) Number of proliferated T cells (left side) and percentage inhibition of T-cell expansion (right side) as in (C). (A–D) Data are representative of 3 independent experiments (*n* = 6). All bar graphs and scatter plots show mean ± SD. Statistics (A, B, C, and D): for comparison of multiple groups, ANOVA or Kruskal–Wallis followed by multiple comparisons was performed. Comparison of two groups (D) was with unpaired *t* test. *P < 0.05, **P < 0.005, and ***P < 0.001. Data used in the generation of this figure can be found in S1 Data. CD, cluster of differentiation; CFSE, carboxyfluorescein succinimidyl ester; COX, cyclooxgenase; ctrl, control; d, day; EP, E Prostanoid; FRC, fibroblastic reticular cell; LN, lymph node; MFI, median fluorescence intensity; ns, not significant; PGE_2_, prostaglandin E_2_; pLN, peripheral LN; SD, standard deviation.

To assess the direct effect of PGE_2_ on T-cell activation and proliferation, naïve T cells were stimulated in the presence of increasing PGE_2_ concentrations. In line with previous data [12], we found that CD8+ and, to a greater extent, CD4+ T-cell proliferation was impaired in the presence of PGE_2_ (Fig 2A) at concentrations similar to those produced by FRCs. Dampened T-cell proliferation was accompanied by reduced CD25 up-regulation on total CD8+ and CD4+ T cells, consistent with a previous report on human T cells [21], whereas CD44 levels were affected mainly in CD8+ T cells (Fig 2B; S2A Fig). Additionally, T-cell CD62L expression was markedly induced upon PGE_2_ treatment (Fig 2B; S2A Fig). To determine whether PGE_2_ is the attenuating factor produced by COX2-expressing FRCs, we used antagonists for the two high-affinity T-cell PGE_2_ receptors EP2 and EP4 [12, 14]. Indeed, CD4+ and CD8+ T-cell proliferation was partially rescued when the EP4 inhibitor was added to the coculture system, to an extent comparable to indomethacin itself (Fig 2C and 2D). In comparison, the effect of the EP2 inhibitor was less pronounced (Fig 2C and 2D). In summary, these in vitro results support the notion that PGE_2_ may be the major COX2-dependent prostanoid released by pLN FRCs dampening T-cell responses by signaling via the EP4 receptor on naïve T cells.

### FRC-derived prostanoids suppress both weak and strong T-cell responses in vitro, while FRC-derived NO mainly dampens strong T-cell responses

**Fig 3:**
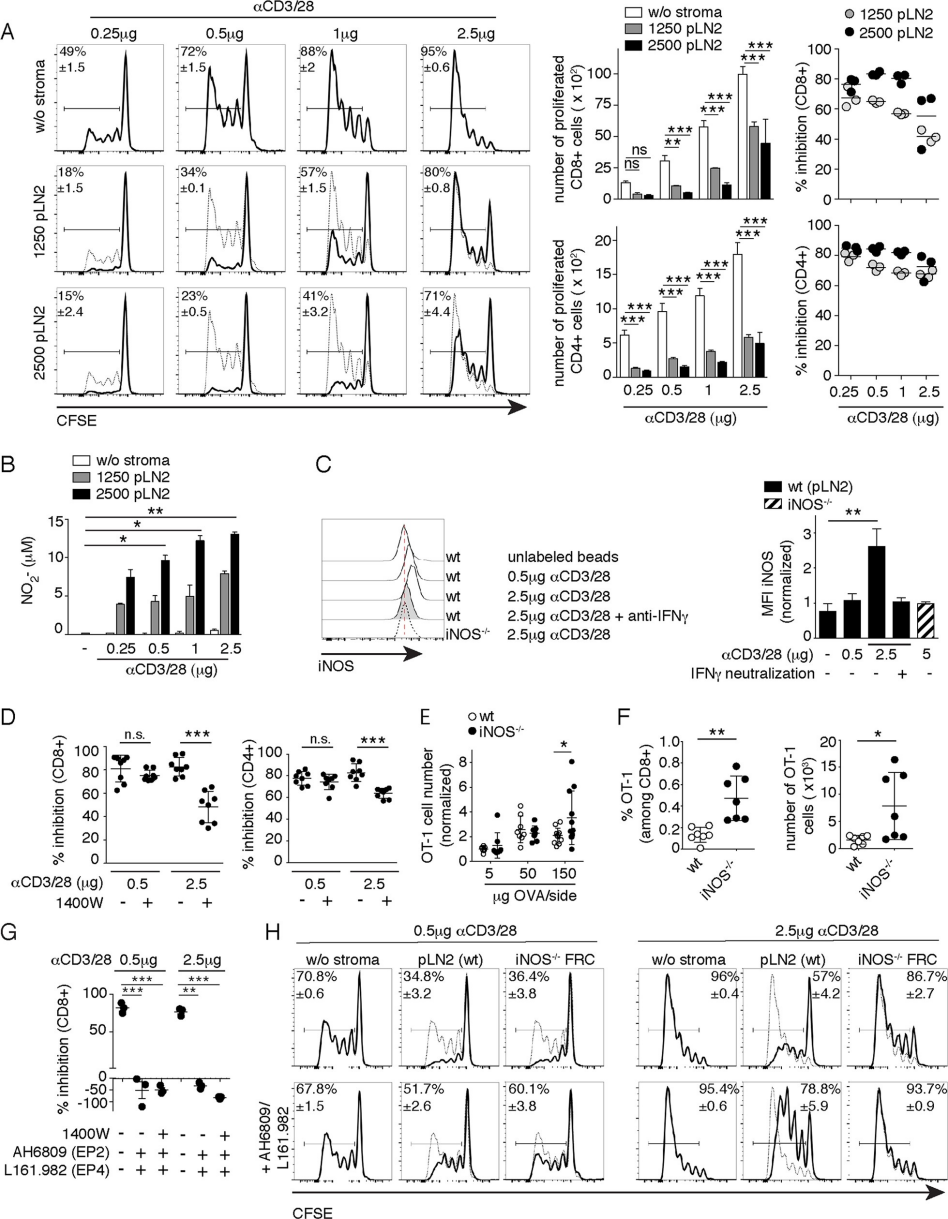
NO released by FRCs dampens mainly strong T-cell responses, while COX2-dependent prostanoids dampen both weak and strong T-cell responses. Flow cytometric analysis of CFSE dilution in CD8+ and CD4+ T cells that were activated with beads coated with indicated concentrations of αCD3/28 and cultured for 3 d ± the indicated numbers of pLN2. (A) Left side: Flow cytometric analysis of CFSE-labeled CD8+ T cells in presence (black line) or absence (thin line) of FRCs. In the middle: bar graphs showing the number of proliferated CD8+ and CD4+ T cells. Right side: scatter plots showing the percentage inhibition of T-cell expansion by FRCs. (B) Griess assay measuring nitrite levels as a surrogate for NO levels in the supernatant of T cell–FRC cocultures described in (A). Data shown in (A) and (B) are representative of 4–5 independent experiments with 3 replicates each. (C) Left side: histograms showing flow cytometric analysis of intracellular iNOS protein expression in pLN FRC cell lines of WT (pLN2) or iNOS^−/−^ origin, cocultured for 2 d with T cells activated with beads coated with the indicated dose of αCD3/28 ± anti-IFNγ (10 μg/ml). Right side: bar graphs showing MFI for iNOS expression in pLN FRC lines normalized to the MFI determined using the iNOS^−/−^ FRC cell line (*n* = 5, pool of 3 independent experiments). (D) T cells activated using anti-CD3/28 beads while being cultured for 3 d ± pLN2 ± iNOS inhibitor 1400W (3 μM) were assessed for the percentage inhibition of T-cell expansion (*n* = 8, pool of 3 independent experiments). (E, F) WT and iNOS^−/−^ mice received OT-1 CD8+ T cells IV, then were immunized SC with OVA/Montanide/Poly(I:C), followed by the flow cytometric analysis of draining pLNs. (E) Scatter plot shows OT-1 T-cell number per dLN on d 4 after immunization with varying doses of antigen. Pool of 2–3 independent experiments (*n* ≥ 8), with data normalized to the average number obtained with the 5 μg OVA condition in WT mice. (F) Scatter plots depict percentages and numbers of OT-1 cells on d 40 after SC immunization with 50 μg OVA/Montanide/Poly(I:C) (*n* = 7; pool of 2 independent experiments). (G–H) Flow cytometric analysis of T cells activated with αCD3/28-coated beads and cultured for 3 d ± pLN2 ± inhibitors: 1400W (for iNOS; 3 μM), AH6809 (for EP2; 5 μM); L161.982 (for EP4; 5 μM). (G) Scatter plot depicting the percentage inhibition of CD8+ T-cell proliferation in presence of the indicated inhibitors (*n* = 3; data representative of 3 independent experiments). (H) CFSE profiles of CD8+ T cells in the presence (thick line) or absence (thin line) of WT pLN2 or iNOS^−/−^ FRC cell line ± AH6809/L161.982 (5 μM each). Data are representative of 3 independent experiments with 2–3 replicates each. Bar graphs and scatter plots showing mean ± SD. Statistics: (A, B, C, and G) ANOVA or Kruskal–Wallis followed by multiple comparisons post-test; (D–F) unpaired *t* test or Mann–Whitney test; *P < 0.05, **P < 0.005, and ***P < 0.001. Data used in the generation of this figure can be found in S1 Data. CD, cluster of differentiation; CFSE, carboxyfluorescein succinimidyl ester; COX, cyclooxygenase; d, day; dLN, draining LN; EP, E Prostanoid; FRC, fibroblastic reticular cell; IFN, interferon; iNOS, inducible nitric oxide synthase; LN, lymph node; MFI, median fluorescence intensity; NO, nitric oxide; ns, not significant; OT-1, ovalbumin-specific CD8+ T cell; OVA, ovalbumin; pLN, peripheral lymph node; Poly(I:C), Polyinosinic-polycytidylic acid; SC, subcutaneous(ly); SD, standard deviation; WT, wild type; w/o, without.

Given that we observed FRCs attenuate T-cell responses via the parallel COX2/PGE_2_ and iNOS/NO pathways, we wished to address why FRCs have two distinct mechanisms to dampen T-cell expansion. Based on the constitutive versus inflammation-induced expression of COX2 and iNOS, respectively, we hypothesized that iNOS-mediated T-cell inhibition may only act during strong T-cell responses when a certain threshold level of inflammation is reached, whereas COX2-mediated T-cell inhibition may act on both strong and weak T-cell responses and thus also affect low-affinity and possibly autoreactive T cells. To test this hypothesis, we used various model systems to mimic strong versus weak T-cell responses. First, we analyzed OVA-specific CD8+ T cell (OT-1) proliferation upon stimulation with bone-marrow–derived (BM)DCs loaded with increasing concentrations of OVA peptide, either of high (N4) or low (V4) OT-1 T-cell receptor (TCR) affinity [22]. Although at least 100 times more V4 than N4 peptide was needed to induce T-cell proliferation and CD44 up-regulation in vitro, we did not find a suitable condition leading to weak T-cell expansion (S2B Fig), in line with previous evidence [22]. When FRCs were added in a setting of strong TCR stimulation due to a high N4 concentration or in settings of weaker TCR stimulation, such as low N4 or high V4 concentration, with BMDCs serving as antigen-presenting cells (APCs), robust inhibition of T-cell proliferation was observed (S2C Fig). Relatively high NO levels were found in the supernatant of all three conditions, indicating that there was sufficient inflammation to induce iNOS expression (S2D Fig). Because BMDCs may contribute NO or Cox-dependent prostanoids in this assay (Fig 1F) [11, 16], we decided to investigate different strengths of T-cell activation in the absence of DCs by using beads loaded with increasing concentrations of αCD3/28 antibodies. In this experimental system, augmenting αCD3/28 concentrations were reflected by a more gradual increase in the expansion of CD8+ and CD4+ T cells (Fig 3A). Adding pLN2 cells in either lower or higher numbers attenuated the T-cell expansion extensively (Fig 3A), with this effect being also visible at the level of CD44 and CD25 up-regulation (S3A Fig). NO levels were augmented by increasing FRC number and TCR strength (Fig 3B) and showed a positive correlation with T-cell inhibition by FRCs (Fig 3A). T-cell–derived IFNγ drives FRC iNOS/NO expression, which then acts in a negative feedback loop to reduce the number of T cells recruited to the response [9–11]. After only 1 day of FRC and T-cell coculture, there was a clear correlation between the strength of TCR activation and the percentage of activated and IFNγ+ CD8+ T cells induced, as well as with intracellular iNOS protein expression and NO levels in the culture supernatant (Fig 3B and 3C, S3B–S3D Fig). Indeed, pharmacological inhibition of iNOS activity with 1400W abolished the suppressive effect by FRCs in the setting of a strong but not weak T-cell stimulation (Fig 3D). These findings support our hypothesis that only strong T-cell stimulation leads to IFNγ secretion by T cells sufficient to induce NO in FRCs, which then limits the number of T cells recruited into the response.

Previously, iNOS-dependent dampening of T-cell expansion in vivo was observed only during strong immune responses [9–11]. To test whether this process is absent during weaker immune responses, WT and iNOS^−/−^ mice having received OVA-reactive CD8+ (OT-1) T cells were immunized subcutaneously (SC) with different doses of OVA/Montanide to mimic different TCR stimulation levels. While the OT-1 T-cell response to low or intermediate antigen doses was comparable between WT and iNOS*-*deficient mice on d 4, the T-cell expansion showed on average a statistically significant increase in response to a high antigen dose only (Fig 3E). Similar to previous studies [10, 11], no differences were observed in the expression of activation marker or in the killing capacity of OT-1 T cells isolated from either WT or iNOS*-*deficient mice (S3E and S3F Fig). The intermediate dose of 50-μg OVA was then used to investigate the development of memory T cells, and we observed a significant increase in memory-phenotype OT-1 T cells in the draining pLNs of iNOS^−/−^ relative to WT mice on d 40 after primary immunization (Fig 3F and S3G Fig). Consistent with the increased number of memory OT-1 T cells, the d 3 response to a secondary immunization in a distant site with a low dose of 5-μg OVA showed a trend towards an increased OT-1 T-cell number in the draining LNs of iNOS^−/−^ relative to WT mice (S3H Fig). Together, these findings suggest that FRCs sense the strength of the T-cell response and produce NO only in the case of a strong primary T-cell response. Interestingly, this inhibitory feedback loop appears to negatively regulate both the generation of effector and memory T cells, thereby limiting the size of the antigen-specific T-cell pool generated.

With iNOS being induced in FRCs during strong primary T-cell responses, we wished to investigate whether the constitutive COX2 pathway might have a complementary role by inhibiting both the strong and weak T-cell responses. Coculture studies revealed an inhibitory role for COX and PGE_2_ receptors EP2/4 in weak but also strong CD8+ and CD4+ T-cell responses (Fig 3G; S3I Fig). This was confirmed by using a newly generated iNOS^−/−^ FRC cell line that did efficiently impair the expansion of weakly but not strongly stimulated T cells (Fig 3H). Also, in this experimental setup, addition of EP2/4 antagonists resulted in the rescue of both weak and strong T-cell responses, suggesting that the COX2/PGE_2_ pathway can dampen T-cell responses independently of the signal strength.

### FRC-specific deletion of COX2 does not affect T-cell homeostasis or clonal expansion in vivo

**Fig 4:**
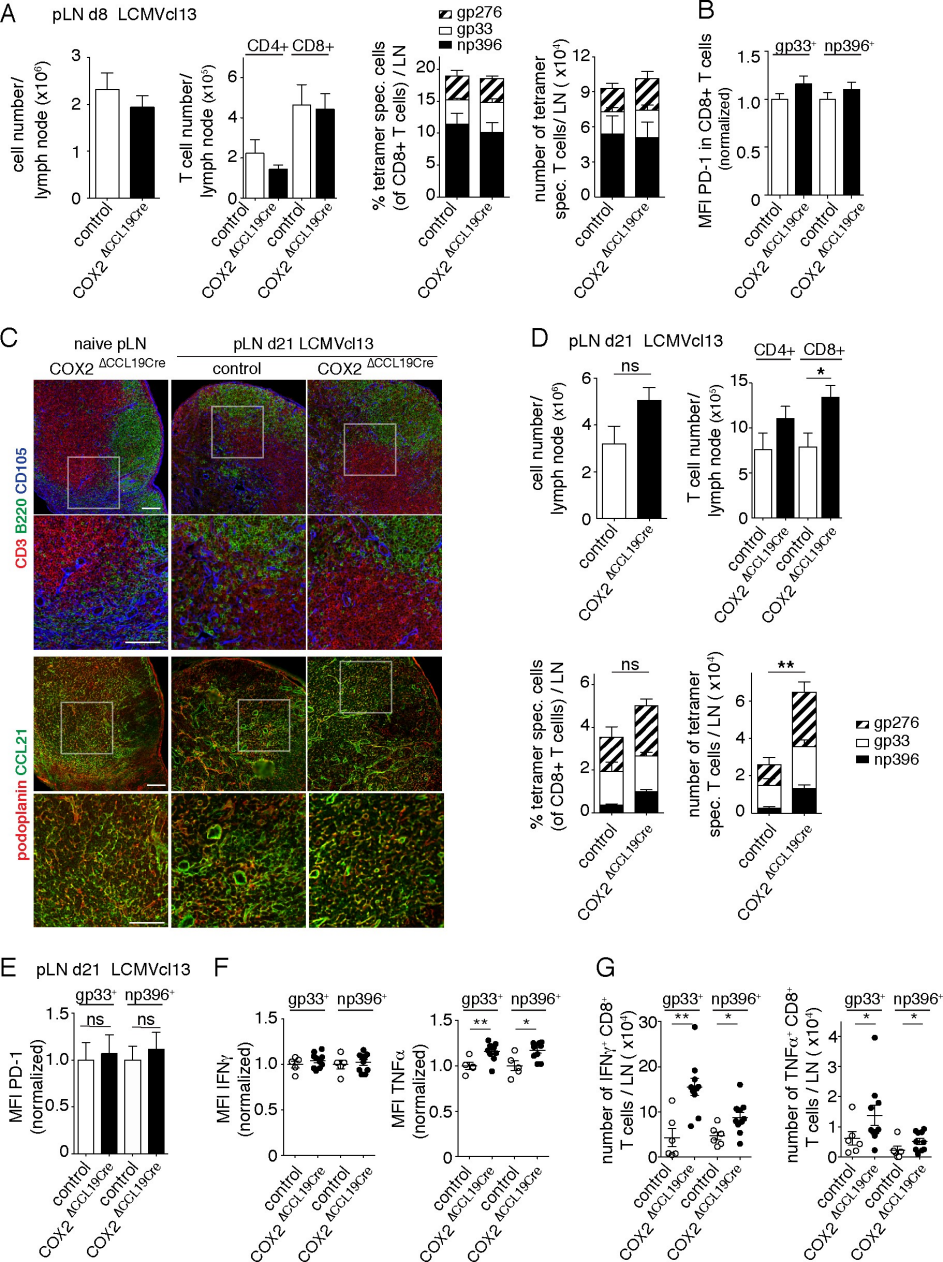
COX2 activity in FRCs dampens chronic T-cell responses during nonresolved LCMV infection in vivo. COX2^ΔCCL19Cre^ and littermate control mice were infected with LCMV clone 13 and analysis performed in draining pLNs at d 8 (A–B) or d 21 (C–G). (A) Bar graphs showing total cell numbers and T-cell numbers per pLN, as well as the percentage and number of LCMV-specific CD8+ T cells in the indicated mice 8 d after LCMV clone 13 infection. (B) MFI of PD-1 expression on gp33- versus np396-specific CD8+ T cells, with results from Cre+ mice being normalized to results obtained in control mice. (A–B: data show a pool of 2 independent experiments with *n* = 9; exception: gp276 tetramer+ cell stainings are based on *n* = 4 in 1 experiment.) (C) Histological analysis of pLNs for the indicated lymphocyte and fibroblast markers. Squares indicate the regions shown enlarged in the row below. Images are representative for 3 mice/genotype. Scale bar, 100 μm. (D) Bar graphs showing total cell numbers and T-cell numbers per pLN, as well as the percentage and number of LCMV-specific CD8+ T cells in the indicated mice. (E) PD-1 expression levels (MFI) on gp33- versus np396-specific CD8+ T cells, normalized to control mice. (F, G) IFNγ and TNFα expression in LCMV-specific CD8+ T cells in pLN after restimulation with gp33 or np396 peptides: MFI (F) versus cell numbers (G); pool of two independent experiments; *n* ≥ 5. (B–E) Bar graphs showing mean ± SEM. Statistics: unpaired *t* test (normally distributed data) or Mann–Whitney test (for not normally distributed data). *P < 0.05, **P < 0.005, and ***P < 0.001. Data used in the generation of this figure can be found in S1 Data. CCL19cre, CCL19 promoter driving Cre recombinase expression; CD, cluster of differentiation; COX, cyclooxygenase; d, day; FRC, fibroblastic reticular cell; gp33, LCMV glycoprotein 33–41 peptide; IFN, interferon; LCMV, lymphocytic choriomeningitis virus; LN, lymph node; MFI, median fluorescence intensity; np396, LCMV nucleoprotein 396–404 peptide; ns, not significant; PD-1, programmed cell death protein 1; pLN, peripheral LN; spec., specific; TNF, tumor necrosis factor.

The absence of reported *nos2*^*flox/flox*^ mice precludes the specific analysis of an in vivo role for iNOS in LN FRCs. Therefore, to test whether LN FRCs can indeed dampen T-cell responses in vivo via COX2, we made mice with FRC-specific COX2 deletion by crossing *ccl19cre* with *ptgs2*^*flox/flox*^ mice [23] (COX2^ΔCCL19cre^). First, we measured the specificity and efficiency of Cre-recombinase–mediated enhanced yellow fluorescent protein (EYFP) expression within different LN cell populations using COX2^ΔCCL19cre^ mice crossed with the Cre-reporter strain *ROSA26*^*eyfp*^ (ROSA26-EYFP^CCL19Cre^). Flow cytometric analysis revealed that 90% of pLN FRCs were EYFP+, whereas less than 15% of endothelial cells and CD31− podoplanin (pdpn)− cells were EYFP+ (S4A Fig). Among the two major FRC subsets, an average of 96% of T-zone FRCs and 76% of medullary FRCs exhibited Cre activity in naïve LNs. This is in line with COX2^ΔCCL19Cre^ mice completely lacking *ptgs2* mRNA expression in T-zone FRCs (S4B Fig). Given that COX2/PGE_2_ expression is constitutive in LN FRCs, we analyzed the hematopoietic and nonhematopoietic cell types in pLN and spleens of naïve COX2^ΔCCL19cre^ mice using flow cytometry and histology; however, no major difference was observed in the size and organization of lymphocyte and FRC populations, nor in the activation status of T cells or the frequency of forkhead box P3 (FoxP3)+ regulatory T cells (Tregs) (S4C–S4I Fig), suggesting that FRC Cox2 is not required to regulate lymphocyte development or homeostasis. To assess whether COX2 activity in FRCs plays a role in regulating T-cell expansion in response to foreign antigens, we infected COX2^ΔCCL19cre^ and control mice with a high dose of LCMV clone 13, which establishes a chronic nonresolving infection. This model system allows analysis of the initial T-cell expansion phase in addition to studying the chronic phase of a viral infection. Notably, PGE_2_ has been recently proposed to directly inhibit T-cell responses to chronic clone 13 infection [18]. COX2^ΔCCL19cre^ mice on d 8 postinfection (p.i.) showed an expansion of LCMV-specific CD8+ T cells within pLNs and spleens that was comparable to WT mice (Fig 4A; S5A and S5B Fig). Splenic LCMV-specific CD8+ T cells in d 8 infected COX2^ΔCCL19cre^ mice showed a trend towards a higher surface expression of programmed cell death protein 1 (PD-1), which was not observed in pLN T cells (Fig 4B, S5C Fig). PD-1 is a negative regulator of T-cell activation in chronic T-cell responses to tumors or chronic infections [24, 25]. Similar findings have been reported recently for LCMV-specific CD8+ T-cell responses in microsomal prostaglandin E synthase-1 (mPges1)- or EP2/4-deficient mice [18], suggesting that this effect in the spleen is probably PGE_2_ driven. To assess the effector function of LCMV-specific CD8+ T cells in these two mouse models on d 8 p.i., we determined their cytokine production as well as the viral clearance by measuring virus titers in the blood as indirect readout of their cytotoxic activity. Indeed, CD8+ T cells isolated from COX2^ΔCCL19cre^ mice exhibited slightly reduced IFNγ and TNFα production upon restimulation with different LCMV peptides (S5D Fig), suggesting COX2 in FRCs has weak proinflammatory effects on the early phase of the T-cell response. However, the viral burden in the blood was similar in both groups (S5E Fig). In line with previous studies [26, 27], our histological analysis showed a major impact of LCMV infection on the LN compartmentalization as well as the function of LN FRCs as a CCL21 source. The FRC network organization, however, was only partially affected in d 8 pLNs (S5F and S5G Fig), in contrast to the previous reports on the extensive destruction of splenic FRC at the peak of the LCMV-specific T-cell response. In this histological analysis, no major differences were observed between the pLNs of COX2^ΔCCL19cre^ and control mice. The observed defects were transient in both mouse models because the LN organization and CCL21 expression were restored by d 21 p.i. (Fig 4C) despite the virus persistence.

### FRC-specific COX2 deletion promotes stronger T-cell responses and better virus control in the chronic phase of LCMV infection

Given the augmented T-cell response previously observed in mPges1^−/−^ mice [18], we wished to assess whether COX2 expression in FRCs was responsible for suppressing chronic T-cell responses. First, we confirmed that LN FRCs are the principal cell type displaying Cre activity on d 19 p.i. with clone 13 virus (S6A Fig), similar to naïve mice. On d 21 p.i., we detected a significant increase in the number of total and LCMV-specific CD8+ T cells in pLNs and, to a lesser extent, in the spleens of COX2^ΔCCL19cre^ mice relative to WT mice, especially for np396 specificities (Fig 4D and S6B Fig). While LCMV-specific cells isolated from COX2^ΔCCL19cre^ mice expressed PD-1 and IFNγ at similar levels to control mice, TNFα expression was increased in cells isolated from pLNs (Fig 4E and 4F), but not spleens (S6C and S6D Fig). Given the strong increase in gp33- and np396-specific T cells in d 21 LNs, the total number of IFNγ+ and TNFα+ T cells was increased by more than 100% in COX2^ΔCCL19cre^ mice (Fig 4G). This increased cytotoxic T lymphocyte (CTL) response translated into an improvement in viral clearance in the blood, pLNs, and spleens of COX2-deficient animals (S6E Fig). Together, these findings demonstrate that COX2 activity within LN FRCs has an important role in suppressing chronic T-cell responses during nonresolved viral infections by down-regulating CTL numbers and function and consequent reduction of viral clearance.

## Discussion

In the current study, we show that FRC lines and ex vivo FRC display a highly active COX2/PGE_2_ pathway that dampens the expansion of in vitro-activated CD8 and CD4 T cells by acting via EP2/4 receptors. This pathway dampens both weak and strong T-cell responses, in contrast to FRC-derived NO, which is produced mainly during strong TCR stimulations, pointing to two distinct inhibitory pathways in FRCs. Using mice selectively lacking COX2 in lymphoid tissue FRCs, we provide direct evidence for an in vivo function of FRC in down-regulating T-cell responses, not during the early phase of an acute CD8 T-cell response but later, during a chronic response. Thus, pLN-FRC–derived COX2 has an anti-inflammatory role on chronic T-cell responses in vivo.

FRCs have been proposed to play dual roles by either promoting or inhibiting adaptive immunity [1, 2], similar to myeloid and T cells. In vitro experiments revealed that FRCs can act as bystander cells to dampen T-cell responses, independently of effects on Tregs or APCs, by sensing IFNγ and TNFα released early during T-cell priming and leading to FRC-derived NO release [9–11]. This latter concept is consistent with in vivo evidence of an exaggerated T-cell response to immunogenic stimuli in iNOS-deficient mice [9–11]. We now extend these findings by showing that iNOS expression and NO release by FRCs in vitro is mainly observed during strong T-cell responses characterized by a marked early IFNγ secretion but not in weaker T-cell responses in which IFNγ levels are low. Because of the lack of iNOS^flox/flox^ mice or suitable chimeric mice [9, 11], this concept could only be tested using iNOS^−/−^ mice in which myeloid cells are also iNOS deficient. Consistent with our in vitro findings, we show here that primary T-cell responses were enhanced in iNOS^−/−^ mice only in response to a high, but not low, antigenic stimulation when investigating the peak of the response. Interestingly, despite a similar response for the d 8 response, the memory T-cell number was increased on d 40 in iNOS^−/−^ relative to WT mice, which translated into a slightly increased recall response, consistent with two earlier reports [28, 29]. We speculate that this regulatory pathway via NO release may only come into effect in acute type I immune responses, which have the potential to damage neighboring cells and therefore need to be tightly controlled. An interesting application of this property of LN FRCs or other mesenchymal stromal/stem cells (MSCs) is their use in settings of acute inflammation, such as transplantation or sepsis, where proinflammatory cytokines are abundant and fibroblasts can unleash their full inhibitory and tissue-preserving function, at least in part by releasing NO [30, 31]. However, both MSCs and LN FRCs have alternative ways of inhibiting T-cell responses because they can express PD-L1, transforming growth factor β (TGFβ), COX2/PGE_2_, and possibly other factors [11, 30, 32–35]. Here, we provide detailed evidence for a second inhibitory pathway in LN FRCs, which is still active in iNOS^−/−^ FRCs and is mediated by the COX2-dependent synthesis of PGE_2_.

We tested the importance of the COX2 pathway specifically in FRCs, both in vitro and in vivo. We observed that COX2 is expressed constitutively in FRCs of naïve LN at levels clearly above those observed for COX1, consistent with a recent report [32]. This is opposite of the usual COX expression pattern, with COX1 being typically constitutively expressed and COX2 being induced during inflammation [12, 16]. LN FRCs maintain this property even ex vivo, including after several weeks of culture, suggesting this is an intrinsic and imprinted feature in murine FRCs [11, 32]. This expression is not much altered upon stimulation by cytokines or Toll-like receptor (TLR)2/4 ligands [11], in contrast to most other cell types, including myeloid cells, which depend upon these signals for COX2 expression [16]. Of note, the gut lamina propria is one of the rare tissues displaying high COX2 expression and thereby an immune microenvironment that attenuates T-cell immunity to the diverse commensals and food antigens. Interestingly, adherent gut stromal cells were identified as major source of COX2, displaying constitutive PGE_2_ expression without need for exogenous stimuli [36, 37]. A recent study revealed that also human FRCs are capable of suppressing T-cell proliferation and differentiation in a COX-dependent manner [35]. In contrast to murine FRCs, both COX1 and COX2 appear to be highly expressed in human FRCs [32, 35].

COX enzymes are the rate-limiting enzymes for the generation of five different prostanoids, including PGE_2_. We decided to focus on this lipid mediator because it is a known negative regulator of T-cell responses and because its high constitutive expression in LN FRCs came as a surprise to us given that LNs are sites of adaptive immune response induction. Remarkably, the expression in ex vivo FRCs was 50-fold above LN macrophages for COX2 transcripts and 10-fold for PGE_2_, suggesting FRCs may be poised to dampen T-cell responses. Indeed, ex vivo FRCs inhibited CD4 and CD8 T-cell activation and expansion in a COX2-dependent manner and to an extent similar to purified PGE_2_ [32]. In addition, blockade of the two major PGE_2_ receptors on T cells, EP2 and EP4, mimicked this effect, providing strong evidence for PGE_2_ being the principal COX2-dependent prostanoid responsible for attenuating T-cell responses in cocultures with pLN FRCs. Importantly, both in vitro and in vivo, COX1 in FRCs was not able to compensate for the COX2 deletion, as demonstrated by the increased T-cell response.

Given the high expression of COX2 and PGE_2_ in pLN FRCs before the start of T-cell priming, we hypothesized this pathway could ensure that responses by nonspecific or weakly activated T cells are avoided [2]. When we varied either the antigen affinity or quantity in the coculture assay, FRC-mediated suppression abolished responses to weaker stimuli, while stronger stimuli overcame these inhibitory effects despite the combined presence of COX2- and iNOS-dependent factors. Importantly, inhibition of COX2 or EP2/4, but not iNOS, again allowed the expansion of weakly responding T cells in FRC-containing cultures. Several findings support the notion that fewer T cells got recruited into the response in presence of the added prostanoid-pathway inhibitors: we observed 1) more undivided cells; 2) a reduced CFSE dilution of those cells that did divide, even when T cells were preactivated in absence of stromal cells; 3) diminished expression of activation markers on undivided but not proliferating T cells; 4) strongly reduced numbers of antigen-specific T cells generated; and 5) fewer effector cells capable of expressing IFNγ, while killing of targets occurred normally. These observations are consistent with earlier reports on PGE_2_ effects on T cells, both by interfering with TCR signaling as well as by deviating in some settings from an IFNγ-driven response [12–14]. For example, when human CD4+ T cells were activated by suboptimal anti-CD3/CD28 stimulation in vitro, most transcriptional changes were ablated by a simultaneous PGE_2_ exposure, leading to strongly reduced cell cycle entry while not altering proapoptotic pathways [20]. Lymphocyte-specific protein tyrosine kinase (Lck), zeta chain of TCR-associated protein kinase 70 (zap70), and Ca flux have been identified as the main targets of negative regulation by PGE_2_ in T cells, with the suppression being surmountable by a stronger stimulation [20]. These findings were recently confirmed in the context of murine T cells cocultured with either murine or human LN FRCs [32]. Our preliminary evidence is consistent with PGE_2_ interfering directly with TCR signaling because enhancing CD28 stimulation (signal 2) or IL-2 did not overcome the attenuating effect by FRCs. Interestingly, NO appears to mediate its inhibitory effect also by interfering with early TCR signaling, such as by nitrosylation of CD3-zeta [38], thereby blocking its phosphorylation sites needed for cell activation. While there is evidence for reciprocal regulation between the iNOS and COX2 pathways [39, 40], the observation that iNOS^−/−^ as well as COX2^−/−^ FRCs [32] can still suppress via the other pathway suggests that at least part of their T-cell inhibitory function is independent. This notion is further supported by the COX2-dependent inhibition of weaker T-cell responses in which iNOS expression is very low. In conclusion, while NO expression by FRCs is transient and limited to strong type I T-cell responses, COX2/PGE_2_ expression in LN FRCs is constitutive and can affect T cells throughout the response and independently of signal strength.

Our findings based on coculture assays raise the question of in vivo relevance, namely, do COX2-dependent mediators derived from FRCs inhibit T-cell responses in settings of homeostasis, immune response, or disease? On one hand, FRCs may participate in maintaining peripheral tolerance by ablating the typically low-affinity responses to self-antigens. So far, we have not observed any obvious autoimmune phenotype in mice lacking COX2 in FRCs (COX2^ΔCCL19cre^) or in all body cells (COX2^−/−^) with mice aged for 20 weeks in our specific-pathogen-free (SPF) animal facility. This is consistent with previous observations in COX2^−/−^ mice [16, 41] as well as with humans treated over months or years with COX inhibitors for which autoimmune side effects have rarely been reported. On the other hand, we hypothesized that FRC-derived PGE_2_ may set a threshold for T-cell activation in response to foreign antigens, either to prevent unnecessary T-cell activation in case of very weak inflammatory stimuli or to focus the response to higher-affinity T cells. Infection with LCMV clone 13 did not reveal any difference in the size of the LCMV-specific CD8+ T cells at the peak of the response, similar to our preliminary evidence from OVA/Montanide vaccination studies investigating the expansion of high-affinity CD4+ OT-2 and CD8+ OT-1 T cells at various time points. This finding may be explained by the weaker attenuation of T-cell responses we observed in presence of ex vivo FRCs compared to the FRC line. However, Cui and colleagues have recently reported an increased OT-1 T-cell expansion at 48 h upon vaccination with OVA-loaded BMDCs in mice lacking an enzymatically active COX2 (COX2^Y385F/Y385F^); notably, this difference was lost 24 h later [32]. Currently, the reason for this discrepancy is unclear but could be based on differences in timing or antigen dose reaching the LNs. It also seems plausible that COX2-dependent processes in non-FRCs may have contributed to the phenotype described by Cui and colleagues because activated macrophages or DCs were also deficient in prostanoid synthesis in their mouse model [32]. Of note, they observed only a delay of the response and not a qualitative or quantitative difference at time points later than 48 h. Nevertheless, these data indicate that PGE_2_ present within the LNs may modulate, in some settings, the T-cell priming or early expansion phase and possibly clonal selection and amplification.

Given the normal T-cell expansion we observed in the early phase of the response to LCMV, we focused our attention on the chronic phase, in which mice deficient in EP2/4 expression in activated T cells or globally deficient in mPges-1 have previously been shown to have an enhanced antiviral CD8+ T-cell response on d 21 post-LCMV infection in number, effector function, and reduction of the “exhaustion” phenotype [18]. That study demonstrated that PGE_2_ acts on d 8 activated virus-specific CD8+ T cells via EP2/4 and reduces the phosphorylation of kinases downstream of TCR signaling such as extracellular signal-regulated kinase (erk) and S6, as well as by dampening IL-2 expression, thereby restricting T-cell survival but not proliferation, presumably without Treg involvement. Interestingly, EP2/4 expression was shown to be increased in PD-1^hi^ T cells on d 21, and inhibiting both COX2 and PD-L1 showed additive effects on T-cell responsiveness [18]. However, this report did not reveal which cells were the main targets of COX2 inhibition. Interestingly, we observed that COX2^ΔCCL19cre^ mice reproduced the findings of Kaech and colleagues, notably the increased numbers of endogenous virus-specific CD8+ T cells of three different specificities, along with their effector function leading to a better virus control without evidence for increased immunopathology [18]. Therefore, we propose that FRCs within T zones of SLOs such as LNs and spleens negatively regulate the survival of chronically activated CD8+ T cells via their constitutive production of PGE_2_, presumably by modulating the chronic TCR signals that drive T-cell exhaustion. Importantly, COX2 inhibition in FRC can partially reverse this effect, both in vitro and in this new in vivo model of FRC-specific COX2 deletion.

COX inhibitors are the most frequently used drugs to prevent inflammation. Therefore, our results suggesting COX2/PGE_2_-expressing FRCs lead to an inhibition rather than enhancement of T-cell responses may seem paradoxical. However, COX-dependent prostanoids and PGE_2_ in particular are well known for their dual role in inflammation and immunity [12–15]. PGE_2_ has been observed to be anti-inflammatory not only in persisting viral infections [18] but also in other chronic diseases, such as various tumor types, in which COX2 and/or PGE_2_ expression by tumors or myeloid-derived suppressor cells have been correlated with suppression and deviation of antitumor T-cell responses, as well as with worse disease outcome [19, 42]. Currently, there are great needs for drugs that can complement the existing checkpoint inhibitors in order to improve the proportion of patients showing clinical benefit. Interestingly, combined inhibition of PD-L1 and COX2 showed additive effects for the recovery of CD8+ T-cell immunity in both models of chronic infection and tumors with apparently limited side effects [18, 19], suggesting COX inhibitors may be used in combination therapy. More specific drugs targeting PGES, PGE_2_, or EP2/4 could be of even greater benefit because they do not affect the other four COX-dependent prostanoid pathways [43]. While inhibition of the COX or PGE_2_ pathway may act on cells residing within the site of chronic inflammation, including tumor cells and cancer associated fibroblasts [44] our study indicates that such inhibitors will also interfere with the anti-inflammatory capacity of FRCs inside SLOs, presumably within T zones, and may permit an improvement of the cancer–immunity cycle [45], namely a revitalization of previously exhausted T-cell clones that can then seed again the sites of inflammation or cancer and maintain an effective immune response.

In summary, we propose that LN FRCs act as a rheostat restraining T-cell responses in at least two different ways, with weak responses being prevented by the omnipresent PGE_2_ and stronger responses being dampened by both PGE_2_ and the inducible NO. During chronic T-cell responses, LN FRCs may have a particularly critical role in restraining them via prostanoid release, presumably in an attempt to resolve the chronic inflammation that can have damaging effects on tissue function. These findings extend and strengthen the concept of suppressive LN stroma that can fine-tune not only the priming and early expansion phase of T cells but also chronic T-cell responses, such as in nonresolved virus infections or cancer.

## Materials and methods

### Ethics statement

All mouse experiments were authorized by the Swiss Federal Veterinary Office (authorization numbers VD1612.3, VD1612.4, and VD3196).

### Mice

C57BL/6J mice were purchased from Envigo (Horst, Netherlands). *Nos2*^−/−^ mice [46] and OT-1 mice [47] were as described. COX2^−/−^, COX2^ΔCCL19Cre^, and ROSA26-EYFP^CCL19Cre^ mice were generated by intercrossing COX2^flox/flox^ [23] with CMVCre [48], CCL19Cre [49], and/or ROSA26-EYFP mice [50], respectively, with Cre− littermate mice used as controls. For experiments, ≥6-week–old mice were used, all with a B6 background. All mouse strains used were bred and maintained in the SPF facility of the University of Lausanne.

### Adoptive cell transfers and immunizations

For adoptive T-cell transfer, 1 × 10^5^ lymphocytes isolated from spleens and pLNs of OT-1 mice (CD45.1+) were transferred IV into recipient mice (CD45.2+). The next day, mice were immunized by SC injections in 6 sites in the flank with 5, 50, or 150 μg OVA (Sigma-Aldrich, St. Louis, MO, USA) diluted in Montanide ISA 25 (25% in PBS; Seppic, Paris, France) containing 50 μg Poly(I:C) (InvivoGen, San Diego, CA, USA; # tlrl-pic). For studying memory responses, 0.2–0.5 × 10^5^ OT-1 cells were transferred IV into recipient mice, followed by an SC immunization at 3 sites of one flank with 50 μg OVA/Montanide containing 25 μg Poly(I:C), with the secondary immunization performed 37 d later with 5 μg OVA/Montanide containing 5 μg Poly(I:C) again injected SC into 3 sites of the opposite flank. The sites of injection into the flank were done in the drainage regions of axillary, brachial, and inguinal LNs, which were then dissected and pooled for the analysis.

### Viral infection

LCMV clone 13 virus stocks were generated according to an established protocol [51]. To obtain a chronic infection 2 × 10^6^ plaque-forming units (PFUs) of LCMV were injected IV and organs collected 8, 19, or 21 d p.i.. To determine viral titers, blood and organ samples were shock frozen on dry ice; tissues were homogenized by bead beating or mashing, and viral titers were determined by a focus-forming assay [51].

### Stromal and hematopoietic cell isolation

In order to investigate LN stromal cells, tissues were collected and digested as described elsewhere [3]. Brief, peripheral LNs (axillary, brachial, and inguinal) were removed and digested for 30 min at 37°C under continuous stirring in DMEM containing 2% FCS, 3 μg/ml collagenase IV (Worthington Biochemical, Lakewood, NJ, USA) and DNAse I (Roche, Basel, Switzerland). Single-cell suspensions of hematopoietic cells were obtained by meshing pLNs and spleens through a 40-μm nylon cell strainer. In spleen samples, erythrocytes were lysed with a Tris-ammonium-chloride–based buffer.

### Cell lines

The WT FRC cell line pLN2 was described previously [11] and stems from naïve pLNs of B6 mice. iNOS^−/−^ FRC cell lines were generated accordingly from pLNs of iNOS^−/−^ mice. All cell lines were cultured in RPMI complete medium (RPMI 1640, 10% FCS, 10 mM HEPES, 50 μM β-Mercaptoethanol, P/S). For experiments, cells were used between passages (p) 24–32 (pLN2) and p23–27 (iNOS^−/−^ FRCs), respectively.

### In vitro T-cell–activation assay using agonistic antibodies

To investigate the effect of FRCs on T-cell activation, cocultures of cells were performed as described previously [11]. Briefly, pLN2 or NOS2^−/−^ FRC lines were plated at 2,500 cells per well of a 96-well plate and, after overnight culture, irradiated with 1,000 rad to prevent their rapid growth. For coculture with ex vivo FRCs, LN stromal cells were isolated by pLN digestion as described above. CD45-negative stromal cells were enriched from cell suspensions using CD45 MicroBeads (Miltenyi Biotec, Bergisch Gladbach, Germany). Stromal cells were seeded in 96-well plates and cultured for 6 days before the addition of T cells in order to get a pure FRC population with a confluency of 70%–80% (FRC purity ≥90%). T cells isolated from spleens and pLNs (axillary, brachial, and inguinal) of WT B6 mice were enriched by panning using antibodies to B220 (RA3-6B2), CD11b (M1/70), and CD11c (N418). These T cells were labeled with 2 μM CFSE (Invitrogen, Carlsbad, CA, USA), resuspended in RPMI complete medium containing 3 ng/ml murine IL-7 (Peprotech, Rocky Hill, NJ, USA), 10 U/ml human IL-2 (Merck Serono, Darmstadt, Germany), and nonessential amino acids (Gibco, Gaithersburg, MD, USA), and plated at 2.5 × 10^5^ cells per well of a 96-well plate, with or without an adherent layer of FRCs. To activate T cells, 1.25 × 10^5^ αCD3/CD28-coupled DynaBeads (Invitrogen) or 2.5 × 10^5^ MACSiBeads (Miltenyi Biotec) coupled with different amounts of αCD3/28 were added to the culture and T-cell proliferation investigated on d 3. Where indicated, inhibitors or the corresponding solvent control were added at d 0 of coculture; for the EP2 and EP4 antagonists, a second bolus was added on d 1. Inhibitor experiments used 10 μM indomethacin (Sigma-Aldrich), 1 μM 1400W dihydrochloride (Sigma-Aldrich), 5 μM L161.982 (EP4 antagonist), and 5 μM AH6809 (EP2 antagonist) (both Cayman Chemical, Ann Arbor, MI, USA). Live cells were counted after 3 d of coculture and analyzed by flow cytometry. Percentage inhibition of T-cell proliferation mediated by FRCs was calculated based on the number of proliferated CD8+ and CD4+ T cells in the presence or absence of FRCs in the coculture.

### In vitro T-cell activation using peptide-loaded BMDCs

As previously described [11], 1 × 10^6^ BMDCs were activated with 0.5 μg/ml LPS (Sigma-Aldrich) for 6 h at 37°C. 2 h after LPS addition, cells were loaded with titrated doses of N4 (SIINFEKL) or V4 (SIIVFEKL) peptide. Lymphocytes were isolated from spleens and pLNs of WT B6 and OT-I mice, erythrocytes were lysed, and CD8 T cells were enriched by panning using antibodies to B220 (RA3-6B2), CD4 (H129.19.6), CD11b (M1/70), and CD11c (N418), followed by labeling with 2 μM CFSE (Invitrogen). Per well of a 24-well plate, 1.96 × 10^6^ WT T cells, 0.04 × 10^6^ CFSE^+^ OT-I T cells, and 1 × 10^4^ BMDCs were cultured alone or in the presence of 1 × 10^4^ irradiated pLN2 FRCs. After 3 d of coculture, cells were harvested and analyzed by flow cytometry.

### Flow cytometry

1–2 × 10^6^ cells were blocked with anti-CD16/32 antibody (2.4G2) and then stained for 40 min at 4°C using the following fluorochrome-coupled antibodies: CD44 (IM7), CD62L (MEL-14), PD-1/CD279 (RMP1-30), TCRβ (H57-597), TNFα (MP6-XT22), IFNγ (XMG1.2), and FoxP3 (FJK-16S) were from eBioscience (Thermo Fisher Scientific, Waltham, MA, USA); CD45pan (30-F11), CD45R/B220 (RA3-6B2), CD8α (53–6.7), CD25 (PC61), and CD31 (390) from Biolegend (San Diego, CA, USA); and CD19 (ID3) and CD157 (BP-3) from BD Biosciences (Franklin Lakes, NJ, USA). CD45.1 (A20), CD4 (H129.19.6), and pdpn (8.1.1) antibodies have been generated in-house. For intracellular staining, cells were fixed and permeabilized using either the BD Cytofix/Cytoperm kit (BD Bioscences) for IFNγ/TNFα or the FoxP3 staining kit (eBioscience).

Endogenous CD8^+^ T cells specific for LCMV were labeled with APC- or PE-conjugated peptide–MHC tetramers (Db/gp33-41, Db/gp276-286, and Db/NP396-404; TC Metrix, Epalinges, Switzerland) for 90 min at 4°C. Dead cells were excluded by marking them with 7-AAD or the Fixable Aqua Dead Cell Stain Kit (both Invitrogen). Samples were acquired on an LSRII Flow Cytometer from BD Biosciences, followed by analysis using FlowJo software (FlowJo LLC, Ashland, OR, USA). Because certain markers were variable in their expression, we have sometimes pooled data sets after normalization to their internal control set at 1. Cell sorting was performed on a FACS-Aria (BD Biosciences) using a 100-μm nozzle. Different LN cell populations were identified as previously described [3]. For RNA isolation, cells were directly sorted into lysis buffer (RNeasy Micro Kit; Qiagen, Hilden, Germany).

### Restimulation and in vitro cytotoxicity assay

Lymphocytes from spleens or pLNs of LCMV infected animals were isolated, and 2 × 10^6^ cells were seeded per well of a 96-well plate. Cells were stimulated in vitro with 1 μM of gp33 (KAVYNFATC) or np396 (FQPQNGQFI) peptide (EMC Microcollections, Tübingen, Germany) for 30 min at 37°C before Brefeldin A (10μg/ml, AppliChem) was added. After another 4 h of incubation at 37°C, cells were harvested, washed, and stained for surface or intracellular epitopes, followed by analysis using flow cytometry. In vitro cytotoxicity assays were performed as previously described [11].

### Histology

Tissues of infected animals were fixed with 2% PFA for at least 4 h, followed by overnight incubation in 30% sucrose before embedding in O.C.T. (Sakura Finetek, Torrance, CA, USA). 8-μm cryosections were generated and immunofluorescence staining performed as described previously [3]. To monitor histological changes in pLN2 or iNOS^−/−^ FRC lines in 8-well chamber slides (Falcon, Milian, Vernier, Switzerland), 7.5 × 10^3^ irradiated FRCs were seeded per chamber (Falcon) and then superseeded by 3.75 × 10^5^ T lymphocytes harvested from spleens and pLNs of WT mice and activated with 3.75 × 10^5^ αCD3/28-coated MACSiBeads. After 2 d of coculture, T cells were removed, and adherent FRCs were stained for microscopic analysis. Cells were fixed with 100% cold acetone, and antibody staining was performed [3]. Images were acquired with a Zeiss Axioplan microscope (Zeiss, Oberkochen, Germany) and treated with Photoshop (Adobe) or ImageJ software, respectively.

The following primary antibodies to murine antigens were used: CD3 (145-2c11), CD31 (GC-51), CD35 (8C12), B220 (RA3-6B2), CD157 (BP 3.4), pdpn (8.1.1), and CD105 (MJ7/18) were produced in-house. Commercial antibodies used were directed to CCL21 (AF457; R&D Systems, Minneapolis, MN, USA), Lyve-1 (103-PA50; RELIATech, Wolfenbüttel, Germany), iNOS (06–573; MilliporeSigma, Burlington, MA, USA), Laminin (L9393, MilliporeSigma), IgG/IgM (315-005-048, Jackson ImmunoResearch, West Grove, PA, USA). Secondary antibodies used were donkey-anti-rabbit IgG-Alexa647, donkey-anti-rat IgG-Alexa488 (Invitrogen), donkey-anti-rabbit IgG-Cy3 and biotin, goat-anti-Armenian hamster IgG biotin and Alexa488, goat-anti-Syrian hamster IgG Cy3, Streptavidin-HRP and Cy3 (all Jackson ImmunoResearch). Antibodies bound to CCL21 were revealed using HRP-conjugated anti-goat IgG (Jackson ImmunoResearch), followed by Cy3-based Tyramide Signal Amplification (Invitrogen Kit #22) according to the manufacturer's instructions. Nuclear labeling was performed using 4′,6-diamidino-2-phenylindole (DAPI; MilliporeSigma).

### Quantitative real-time PCR

To investigate the genetic profile of stroma versus hematopoietic cell enriched tissue fractions, spleens or pLNs were meshed through a 40-μm filter [4]. The nonsoluble stroma remaining in the filter was harvested directly in TRIzol (Ambion; Life Technologies, Carlsbad, CA, USA), whereas the soluble hematopoietic cells were centrifuged and then resuspended in TRIzol. These TRIzol samples were homogenized by bead beating and the RNA extracted [3]. RNA of sorted cells was isolated using RNeasy Micro Kit (Qiagen). Reverse transcription and quantitative real-time PCR was performed as described previously [3]. Efficiency-corrected RNA expression was normalized to the expression of the two housekeeping genes *hprt* and *tbp*. Sequences of primer pairs used are as follows:

*inos* (NOS2): Fwd, 5′- gtt ctc agc cca aca ata caa ga -3′ and Rev, 5′- gtg gac ggg tcg atg tca c-3′

*ptgs1* (COX1): Fwd, 5′- cca gag tca tga gtc gaa gga g -3′ and Rev, 5′- CCT GGT TCT GGC ACG GAT AG -3′

*ptgs2* (COX2): Fwd, 5′-AAT TAC TGC TGA AGC CCA CC-3′ and Rev, 5′-CTT CCC AGC TTT TGT AAC CAT-3′

*ptges1*: Fwd, 5′-GCA CAC TGC TGG TCA TCA AG-3′ and Rev, 5′- ACG TTT CAG CGC ATC CTC-3′

*ptges2*: Fwd, 5′- CAG GTG GTG GAG GTG AAT CC-3′ and Rev, 5′- CTG CCC TGA AAC CAG GTA GG-3′

*ptges3*: Fwd, 5′- cga att ttg acc gtt tct ctg-3′ and Rev, 5′- tga atc atc atc tgc tcc atc t-3′

*hprt*: Fwd, 5′- gttggatatgcccttgac-3′ and Rev, 5′- agg act aga aca cct gct-3′

*tbp*: Fwd, 5′- CCT TCA CCA ATG ACT CCT ATG AC-3′ and Rev, 5′- CAA GTT TAC AGC CAA GAT TCA C-3′

### PGE_2_ and nitrite detection

PGE_2_ levels were determined in culture supernatants using the PGE_2_ ELISA Kit (Cayman Chemical) according to the manufacturer’s instructions. The level of nitrite (NO_2_−) in cell culture supernatants was measured using the Griess assay [11].

### Statistical analysis

Statistical analyses were performed with Prism (version 6.0b, 7.0d and 8.1.1, GraphPad Software).

Unpaired *t* test (normally distributed data) or Mann–Whitney test (for not normally distributed data) were used to compare two data sets. To compare multiple groups, one-way ANOVA or Kruskal–Wallis nonparametric tests were performed, followed by a post hoc test. *P* values < 0.05 were considered to be statistically significant.

## Supporting information

S1 Data Raw numbers used to construct primary and supplemental figures. Also available at DOI: 10.5281/zenodo.3256348.
(XLSX)

S1 Fig Activation of CD4+ T cells is dampened by iNOS and COX2 activity in pLN FRCs in a similar way as CD8+ T cells. (A–B) T cells were activated with αCD3/28 DynaBeads and cultured for 3 d in the absence (white bars) or presence of pLN2 (black bars) ± 1400W (3 μM) ± indomethacin (10 μM). Flow cytometric analysis showing the MFI of CD44 and CD25 on proliferated CD8+ T cells (A) and total or proliferated CD4+ T cells (B). Data pooled from four independent experiments, *n* = 10. (C, D) CFSE-labeled T cells were either activated with αCD3/28 DynaBeads and cultured ± pLN2 or preactivated for 24 h with αCD3/28 DynaBeads followed by 2 d culture + pLN2. (C) Scatter plot showing percentage inhibition of CD4+ T cells mediated by pLN2. (D) MFI of CD44 and CD25 of CD4+ T cells cocultured with pLN2 and normalized to the MFI of CD4+ T cells cultured without pLN2. Data shown in (C) and (D) represent a pool of 3 independent experiments (*n* = 9). (E) RT-qPCR analysis for *Nos2*, *ptgs2*, and *ptgs1* transcript levels in pLN2 cells that were left unstimulated or stimulated for 7 h with 10 ng/ml of both IFNγ and TNFα or with 0.5 μg/ml LPS (*n* = 3). (F–G) RT-qPCR analysis of the soluble (lymphocyte-enriched) and nonsoluble (stroma-enriched) fractions of pLNs and spleens of naïve WT mice (*n* = 4) for transcripts of *ptgs2/1* (F) or *ptges1/2/3* (G). All bar graphs indicate the mean ± SD. Statistics: (A), (B), (C), (F), and (G) using unpaired *t* test or Mann–Whitney, respectively. (D and E) ANOVA or Kruskal–Wallis, followed by multiple comparisons test. *P < 0.05, **P < 0.005, and ***P < 0.001. Data used in the generation of this figure can be found in S1 Data. CD, cluster of differentiation; CFSE, carboxyfluorescein succinimidyl ester; COX, cyclooxygenase; d, day; IFN, interferon; iNOS, inducible nitric oxide synthase; LN, lymph node; LPS, lipopolysaccharide; MFI, median fluorescence intensity; pLN, peripheral LN; *ptgs*, prostaglandin-endoperoxide synthase; RT-qPCR, reverse transcription followed by a quantitative polymerase chain reaction; SD, standard deviation; TNF, tumor necrosis factor; WT, wild type.
(TIF)

S2 Fig LN FRCs can dampen the expansion of strongly or weakly activated CD8+ T cells. Flow cytometric analysis of CFSE-labeled T cells activated in vitro for 3 d, either polyclonally with αCD3/28-coated beads (A) or with peptide antigens presented by BMDCs (B–C). (A) T cells cultured in absence of pLN2 but with the indicated concentrations of PGE_2_ (black bars) or solvent control (white bars), respectively. Shown is the MFI of CD44, CD25, and CD62L on total CD4+ T cells, normalized to the MFI of untreated cells in order to see the fold difference in the expression level upon treatment. *n* = 6; pool of 3 independent experiments. (B) CFSE-labeled OT-1 CD8+ T cells were mixed in a ratio of 1:50 with WT T cells and cultured with LPS-activated BMDCs pulsed with the indicated concentrations of OVA peptides of high affinity (N4) or low affinity (V4) for the OT-1 receptor, ± pLN2 FRCs. OT-1 cell proliferation or activation (B, C) or nitrite levels (D) were assessed after 3 d of culture. (B) CFSE profiles (left side), numbers (middle panel), and CD44 expression levels (right panel) of OT-1 T cells activated in the absence of the pLN2 FRC line. Data are representative of 2 independent experiments performed in duplicates. (C) CFSE profile of OT-1 T cells cultured in the absence (thin line) or presence (black line) of pLN2 FRCs. Scatter dot plot depicts the percentage inhibition of OT-1 T-cell proliferation by FRCs. (D) Bar graphs showing nitrite (NO_2_−) levels found in the supernatant of the cocultures shown in (C). Data in (C) and (D) represent a pool of 2 independent experiments; *n* ≥ 4. Statistics: (A and D) unpaired *t* test or Mann–Whitney test was performed. *P < 0.05, **P < 0.005, and ***P < 0.001. Data used in the generation of this figure can be found in S1 Data. BMDC, bone-marrow–derived dendritic cell; CD, cluster of differentiation; CFSE, carboxyfluorescein succinimidyl ester; d, day; FRC, fibroblastic reticular cell; LN, lymph node; LPS, lipopolysaccharide; MFI, median fluorescence intensity; OT-1, ovalbumin-specific CD8+ T cell; PGE_2_, prostaglandin E_2_; pLN, peripheral LN; WT, wild type.
(TIF)

S3 Fig The magnitude of iNOS-mediated T-cell inhibition correlates with the strength of T-cell activation and early IFNγ production but does not impact effector function of proliferating cells. (A–D) CD8+ and CD4+ T cells were activated with the indicated amount of αCD3/28-coated onto MicroBeads ± pLN2 FRCs. (A) MFI of CD8+ T cells cultured for 3 d ± pLN2 in the indicated numbers. Data are representative of 4–5 independent experiments with 3 replicates each. (B) The frequency of IFNγ-producing CD8+ T cells were investigated after 1 d of coculture. One representative out of 2–3 independent experiments is shown, with at least 2 replicates in each experiment. (C) Histological analysis of d 2 cocultures containing FRCs and activated CD8+ and CD4+ T cells for iNOS protein expression in pdpn+ FRCs. DAPI highlights cell nuclei. Some cultures contained neutralizing anti-IFNγ antibodies. Scale bar, 100 μm. Shown photos are representative of 3 independent experiments. (D) WT (pLN2) and iNOS^−/−^ FRC cell lines were cocultured with activated T cells ± anti-IFNγ antibodies, and nitrite levels measured in the SN of d 2 cultures using the Griess assay. Scatter plot showing 1 representative out of 3 independent experiments. (E–G) WT or iNOS^−/−^ mice that had received OT-1 CD8+ T cells IV were immunized SC with OVA/Montanide and the draining pLNs investigated on d 4 after immunization. (E) Bar graphs depict the MFI of CD25 expression on OT-1 T cells isolated from WT versus iNOS^−/−^ mice immunized with the indicated concentrations of OVA (*n* ≥ 8, pool of 2–3 independent experiments). (F) Cytotoxic capacity of OT-1 T cells isolated from draining pLNs of WT and iNOS^−/−^ mice immunized SC 4 d earlier with 150 μg OVA/Montanide/Poly(I:C). Shown is the percentage of target cell lysis with the indicated E:T ratios for one representative (*n* = 3) out of 2 independent experiments. (G) Memory phenotype of CD44+ OT-1 T cells (CD45.1 + CD8α+) in draining pLNs on d 40 after SC immunization with 50 μg OVA/Montanide/Poly(I:C). *n* = 7. (H) The indicated mice received a primary immunization SC in the left flank with 50 μg OVA in Montanide/Poly(I:C) adjuvant and were challenged on d 37 after primary immunization with 5 μg OVA/Montanide/Poly(I:C) SC in the right flank, with LNs draining the second site being investigated 3 d later. Bar graphs depict numbers and percentages of OT-1 cells in WT and iNOS^−/−^ mice, respectively (*n* ≥ 10; pool of 3 independent experiments). (I) Cocultures of T cells activated with beads coated with either 0.5 or 2.5 μg CD3/28 antibody and cultured for 3 d ± pLN2 FRCs ± indicated inhibitors (1400W [3 μM], AH6809 [5 μM], L161.982 [5 μM]). Scatter plot depicts the percentage inhibition of CD4+ T-cell expansion mediated by pLN2 (*n* = 3; show 1 representative of 3 independent experiments). Statistics: (D, E, and I) ANOVA or Kruskal–Wallis followed by multiple comparisons post-test; (H) unpaired *t* test or Mann–Whitney. *P < 0.05, **P < 0.005, and ***P < 0.001. Data used in the generation of this figure can be found in S1 Data. CD, cluster of differentiation; d, day; E:T, Effector:Target; FRC, fibroblastic reticular cell; IFN, interferon; iNOS, inducible nitric oxide synthase; LN, lymph node; MFI, median fluorescence intensity; OT-1, ovalbumin-specific CD8+ T cell; OVA, ovalbumin; pLN, peripheral LN; pdpn, podoplanin; Poly(I:C), Polyinosinic-polycytidylic acid; SC, subcutaneous(ly); SN, supernatant; WT, wild type.
(TIF)

S4 Fig Deletion of COX2 expression specifically in FRCs does not alter T-cell homeostasis or pLN structure. Characterization of pLNs and spleens of naïve mice genetically lacking COX2 expression specifically in FRCs (COX2^ΔCCL19Cre^) versus their littermate Cre− mice (called “controls”). (A) CCL19Cre activity was investigated in pLNs of naïve COX2^ΔCCL19Cre^ ROSA26-EYFP^CCL19Cre^ reporter mice. Histograms showing EYFP expression in different nonhematopoietic cell types (CD45−): CD31− pdpn− DN cells, CD31+ pdpn− BECs, CD31+ pdpn+ LECs, and CD31− pdpn+ FRCs in EYFP reporter (solid black line) compared to control Cre− mice (gray shading). FRCs were further divided into CD157− MedRCs and CD157+ TRCs. *n* = 3, representative data of 2 independent experiments. (B–G) Characterization of naïve pLNs of COX2^ΔCCL19Cre^ mice. (B) Transcript levels of *ptgs2* were analyzed in sorted TRCs (CD45− CD35− CD31− pdpn+ CD157+). *n* = 2–3; each sample represents a pool of 2–3 mice. Transcript levels below the detection limit or nonspecific transcripts are indicated as white circles on the x-axis. (C) Scatter plots showing total cell numbers (left side) or CD4+ and CD8+ T-cell numbers (middle) in digested naïve pLNs or representative histograms (right side) showing the percentage of activated CD8+ and CD4+ T cells by gating on CD25^high^ or CD62L^low^ cells in samples derived from Cre+ (thick line) or Cre− littermates (dashed line with gray shading). (D, E) Scatter plots showing percentage of FoxP3+ CD25+ Treg among CD4+ T cells (D) and total FRC and EC numbers (E). (F) Immunofluorescence microscopy analysis of labeled pLN sections. Localization of T and B cells, as well as antibody staining for different stromal cell types or their products, is shown. Data are representative for 2 independent experiments investigating 3 mice per genotype. Scale bar, 100 μm. (G) Scatter plots showing CD19+ B-cell numbers in pLN. (H–I) Flow cytometric characterization of naïve spleens of COX2^ΔCCL19Cre^ mice. Shown are scatter plots depicting total cell numbers, lymphocyte numbers (H), and Treg cell numbers (I). Data in (C), (D), (E), (G), (H), and (I) show 1 (*n* = 4) representative out of 2 (D, I) and 3 (C), (E), (G), and (H) independent experiments. All bar graphs and scatter plots showing mean ± SD. Statistics (C, D, E, G, H, and I): Unpaired *t* test or Mann–Whitney test. *P < 0.05, **P < 0.005, and ***P < 0.001. Data used in the generation of this figure can be found in S1 Data. BEC, blood endothelial cell; CCL19cre, CCL19 promoter driving Cre recombinase expression; CD, cluster of differentiation; COX, cyclooxygenase; DN, double negative; EC, endothelial cell; EYFP, enhanced yellow fluorescent protein; FoxP3, forkhead box P3; FRC, fibroblastic reticular cell; LEC, lymphatic endothelial cell; LN, lymph node; MedRC, Medullary FRC; pLN, peripheral LN; pdpn, podoplanin; *ptgs*, prostaglandin-endoperoxide synthase; ROSA, *rosa26* promoter; SD, standard deviation; TRC, T-zone FRC; Treg, regulatory T cell.
(TIF)

S5 Fig COX2 deletion in FRCs does not alter the cellular composition and organization of pLNs and spleens 8 d post-LCMV clone 13 infection. COX2^ΔCCL19Cre^ and Cre− littermate mice were infected with 2 × 10^6^ PFU LCMV clone 13 and the spleens and pLNs analyzed on d 8 p.i.. Bar graphs showing (A) total cell numbers and T-cell numbers as well as (B) frequencies and cell numbers of LCMV-specific CD8+ T cells (specific for three viral peptides) in the spleen. Shown is a pool of 2 independent experiments; *n* = 9, except for gp276 tetramer+ cells with *n* = 4 of 1 experiment. (C) MFI of PD-1 on splenic LCMV-specific CD8+ T cells, with cells from Cre+ mice normalized to those from Cre− mice. (D) Frequencies of splenic IFNγ- and TNFα-expressing LCMV-specific T cells determined after short restimulation with gp33 or np396 peptides, respectively. Scatter plot showing normalized frequencies of LCMV-specific cells of Cre+ compared to Cre− controls. Data in (C) and (D) show a pool of 2 independent experiments; *n* ≥ 5. (E) Box and whisker plots showing viral titer in the blood (*n* ≥ 7). (F) Immunofluorescence microscopy analysis of labeled pLN sections from naïve or d 8 LCMV-clone-13–infected mice of the indicated genotype. Representative images from 3 mice/genotype are depicted. Scale bar, 100 μm. (G) Stitched images of labeled pLN sections described in (F). Scale bar, 200 μm. Bar graphs and scatter plots showing mean ± SEM. Statistics: unpaired *t* test or Mann–Whitney test. *P < 0.05, **P < 0.005, and ***P < 0.001. Data used in the generation of this figure can be found in S1 Data. CCL19cre, CCL19 promoter driving Cre recombinase expression; CD, cluster of differentiation; COX, cyclooxygenase; d, day; FRC, fibroblastic reticular cell; gp33, LCMV glycoprotein 33–41 peptide; IFN, interferon; LCMV, lymphocytic choriomeningitis virus; LN, lymph node; MFI, median fluorescence intensity; np396, LCMV nucleoprotein 396–404 peptide; PFU, plaque-forming unit; pLN, peripheral LN; p.i., postinfection; TNF, tumor necrosis factor.
(TIF)

S6 Fig COX2 deletion in FRCs leads to an increased virus-specific CD8+ T-cell response during the chronic phase of LCMV clone 13 infection. COX2^ΔCCL19Cre^ mice were infected with LCMV clone 13 and pLNs and spleens analyzed during the chronic phase at d 19–21 after infection, using flow cytometry (A–D) or plaque-forming assay (E). (A) pLN of COX2^ΔCCL19Cre^ ROSA26-EYFP^CCL19Cre^ mice were digested enzymatically and analyzed on d 19 p.i.. Cre activity was investigated by measuring EYFP levels in different cell types using flow cytometry. Dot plot shows gating strategy to distinguish CD31− pdpn− DN cells, CD31+ pdpn− BECs, CD31+ pdpn+ LECs, and CD31− pdpn+ FRCs after pregating on CD45− CD35− nonhematopoietic cells. The FRC population was further subdivided into CD157− MedRCs and CD157+ TRCs. The histograms show the frequency of EYFP-expressing cells among different stromal cell subsets, with the cells from Cre− mice shown with gray shading and those from Cre+ mice in black lines. One out of two representative experiments is shown (*n* = 3). (B–D) The spleens of COX2^ΔCCL19Cre^ and control mice were analyzed on d 21 post-clone 13 infection for the frequency and number of LCMV-specific CD8+ T cells (B), for the MFI of intracellular IFNγ or TNFα levels in virus-specific CD8+ T cells (C), or for the number of IFNγ- or TNFα-expressing splenic CD8+ T cells (D; normalized to controls) after restimulation with gp33 or np396 peptides, respectively. (C, D) Pool of 3 independent experiments; *n* ≥ 6. (B–D) Bar graphs and scatter plots showing mean ± SEM. (E) Box and whisker plots showing viral titers in the blood, pLNs, and spleens of d 21 LCMV-clone-13–infected Cre− and Cre+ mice. Statistics: unpaired *t* test or Mann–Whitney test. *P < 0.05, **P < 0.005, and ***P < 0.001. Data used in the generation of this figure can be found in S1 Data. BEC, blood endothelial cell; CCL19cre, CCL19 promoter driving Cre recombinase expression; CD, cluster of differentiation; COX, cyclooxygenase; d, day; DN, double negative; EYFP, enhanced yellow fluorescent protein; FRC, fibroblastic reticular cell; IFN, interferon; LCMV, lymphocytic choriomeningitis virus; LEC, lymphatic endothelial cell; LN, lymph node; MedRC, Medullary FRC; MFI, median fluorescence intensity; pLN, peripheral LN; pdpn, podoplanin; ROSA, *rosa26* promoter; TNF, tumor necrosis factor; TRC, T-zone FRC.
(TIF)

## Abbreviations

APC: antigen-presenting cell
BMDC: bone-marrow–derived DC
CCL19: C-C motif ligand 19 chemokine
CCL19cre: CCL19 promoter driving Cre recombinase expression
CD: cluster of differentiation
CFSE: carboxyfluorescein succinimidyl ester
COX: cyclooxygenase
CTL: cytotoxic T lymphocyte
d: day
DC: dendritic cell
dLN: draining lymph node
EP: E Prostanoid
erk: extracellular signal-regulated kinase
EYFP: enhanced yellow fluorescent protein
FoxP3: forkhead box P3
FRC: fibroblastic reticular cell
GM-CSF: Granulocyte-Macrophage Colony-Stimulating Factor
gp33: LCMV glycoprotein 33–41 peptide
IFN: interferon
IL: interleukin
iNOS: inducible nitric oxide synthase
Lck: lymphocyte-specific protein tyrosine kinase
LCMV: lymphocytic choriomeningitis virus
LN: lymph node
LPS: lipopolysaccharide
MFI: median fluorescence intensity
MHC: major histocompatibility complex
mPges1: microsomal prostaglandin E synthase-1
MSC: mesenchymal stromal/stem cell
Mø: macrophage
Nos2: gene coding iNOS
np396: LCMV nucleoprotein 396–404 peptide
NSAID: nonsteroidal anti-inflammatory drug
OT-1: ovalbumin-specific CD8+ T cell
OVA: ovalbumin
p: passage
pdpn: podoplanin
PD-1: programmed cell death protein 1
PFU: plaque-forming unit
PGE_2_: prostaglandin E_2_
pLN: peripheral LN
Poly(I:C): Polyinosinic-polycytidylic acid
ptges: prostaglandin E synthase
ptgs: prostaglandin-endoperoxide synthase
p.i.: postinfection
ROSA: *rosa26* promoter
RT-qPCR: reverse transcription followed by a quantitative polymerase chain reaction
SC: subcutaneous(ly
SLO: secondary lymphoid organ
SPF: specific-pathogen free
SD: standard deviation
TCR: T-cell receptor
TGFβ: transforming growth factor β
TNF: tumor necrosis factor
TLR: Toll-like receptor
Treg: regulatory T cell
WT: wild type
zap70: zeta chain of TCR associated protein kinase 70

## References

[1] Malhotra D, Fletcher AL, Turley SJ. Stromal and hematopoietic cells in secondary lymphoid organs: partners in immunity. Immunological reviews. 2013;251(1):160–76. 10.1111/imr.12023 23278748 PMC3539229

[2] Siegert S, Luther SA. Positive and negative regulation of T cell responses by fibroblastic reticular cells within paracortical regions of lymph nodes. Frontiers in immunology. 2012;3:285 10.3389/fimmu.2012.00285 22973278 PMC3438460

[3] Link A, Vogt TK, Favre S, Britschgi MR, Acha-Orbea H, Hinz B, et al Fibroblastic reticular cells in lymph nodes regulate the homeostasis of naive T cells. Nature immunology. 2007;8(11):1255–65. 10.1038/ni1513 . 17893676

[4] Luther SA, Tang HL, Hyman PL, Farr AG, Cyster JG. Coexpression of the chemokines ELC and SLC by T zone stromal cells and deletion of the ELC gene in the plt/plt mouse. Proceedings of the National Academy of Sciences of the United States of America. 2000;97(23):12694–9. 10.1073/pnas.97.23.12694 . 11070085 PMC18826

[5] Bajenoff M, Egen JG, Koo LY, Laugier JP, Brau F, Glaichenhaus N, et al Stromal cell networks regulate lymphocyte entry, migration, and territoriality in lymph nodes. Immunity. 2006;25(6):989–1001. 10.1016/j.immuni.2006.10.011 . 17112751 PMC2692293

[6] Katakai T, Hara T, Lee JH, Gonda H, Sugai M, Shimizu A. A novel reticular stromal structure in lymph node cortex: an immuno-platform for interactions among dendritic cells, T cells and B cells. Int Immunol. 2004;16(8):1133–42. 10.1093/intimm/dxh113 . 15237106

[7] Fletcher AL, Lukacs-Kornek V, Reynoso ED, Pinner SE, Bellemare-Pelletier A, Curry MS, et al Lymph node fibroblastic reticular cells directly present peripheral tissue antigen under steady-state and inflammatory conditions. The Journal of experimental medicine. 2010;207(4):689–97. Epub 2010/03/24. jem.20092642 [pii] 10.1084/jem.20092642 20308362 PMC2856033

[8] Dubrot J, Duraes FV, Potin L, Capotosti F, Brighouse D, Suter T, et al Lymph node stromal cells acquire peptide-MHCII complexes from dendritic cells and induce antigen-specific CD4(+) T cell tolerance. The Journal of experimental medicine. 2014;211(6):1153–66. 10.1084/jem.20132000 24842370 PMC4042642

[9] Khan O, Headley M, Gerard A, Wei W, Liu L, Krummel MF. Regulation of T cell priming by lymphoid stroma. PLoS ONE. 2011;6(11):e26138 10.1371/journal.pone.0026138 22110583 PMC3215700

[10] Lukacs-Kornek V, Malhotra D, Fletcher AL, Acton SE, Elpek KG, Tayalia P, et al Regulated release of nitric oxide by nonhematopoietic stroma controls expansion of the activated T cell pool in lymph nodes. Nature immunology. 2011;12(11):1096–104. 10.1038/ni.2112 21926986 PMC3457791

[11] Siegert S, Huang HY, Yang CY, Scarpellino L, Carrie L, Essex S, et al Fibroblastic reticular cells from lymph nodes attenuate T cell expansion by producing nitric oxide. PLoS ONE. 2011;6(11):e27618 10.1371/journal.pone.0027618 22110693 PMC3215737

[12] Kalinski P. Regulation of immune responses by prostaglandin E2. Journal of immunology. 2012;188(1):21–8. 10.4049/jimmunol.1101029 22187483 PMC3249979

[13] Harris SG, Padilla J, Koumas L, Ray D, Phipps RP. Prostaglandins as modulators of immunity. Trends in immunology. 2002;23(3):144–50. . 11864843 10.1016/s1471-4906(01)02154-8

[14] Narumiya S. Prostanoids and inflammation: a new concept arising from receptor knockout mice. Journal of molecular medicine. 2009;87(10):1015–22. 10.1007/s00109-009-0500-1 . 19609495

[15] Sreeramkumar V, Fresno M, Cuesta N. Prostaglandin E2 and T cells: friends or foes? Immunology and cell biology. 2012;90(6):579–86. 10.1038/icb.2011.75 21946663 PMC3389798

[16] Rocca B, FitzGerald GA. Cyclooxygenases and prostaglandins: shaping up the immune response. International immunopharmacology. 2002;2(5):603–30. . 12013502 10.1016/s1567-5769(01)00204-1

[17] Baglole CJ, Ray DM, Bernstein SH, Feldon SE, Smith TJ, Sime PJ, et al More than structural cells, fibroblasts create and orchestrate the tumor microenvironment. Immunological investigations. 2006;35(3–4):297–325. 10.1080/08820130600754960 . 16916756

[18] Chen JH, Perry CJ, Tsui YC, Staron MM, Parish IA, Dominguez CX, et al Prostaglandin E2 and programmed cell death 1 signaling coordinately impair CTL function and survival during chronic viral infection. Nature medicine. 2015;21(4):327–34. 10.1038/nm.3831 25799228 PMC4505619

[19] Zelenay S, van der Veen AG, Bottcher JP, Snelgrove KJ, Rogers N, Acton SE, et al Cyclooxygenase-Dependent Tumor Growth through Evasion of Immunity. Cell. 2015;162(6):1257–70. 10.1016/j.cell.2015.08.015 26343581 PMC4597191

[20] Chemnitz JM, Driesen J, Classen S, Riley JL, Debey S, Beyer M, et al Prostaglandin E2 impairs CD4+ T cell activation by inhibition of lck: implications in Hodgkin's lymphoma. Cancer research. 2006;66(2):1114–22. 10.1158/0008-5472.CAN-05-3252 . 16424048

[21] Rincon M, Tugores A, Lopez-Rivas A, Silva A, Alonso M, De Landazuri MO, et al Prostaglandin E2 and the increase of intracellular cAMP inhibit the expression of interleukin 2 receptors in human T cells. European journal of immunology. 1988;18(11):1791–6. Epub 1988/11/01. 10.1002/eji.1830181121 . 2849551

[22] Zehn D, Lee SY, Bevan MJ. Complete but curtailed T-cell response to very low-affinity antigen. Nature. 2009;458(7235):211–4. 10.1038/nature07657 19182777 PMC2735344

[23] Ishikawa TO, Herschman HR. Conditional knockout mouse for tissue-specific disruption of the cyclooxygenase-2 (Cox-2) gene. Genesis. 2006;44(3):143–9. 10.1002/gene.20192 . 16496341

[24] Shin H, Wherry EJ. CD8 T cell dysfunction during chronic viral infection. Current opinion in immunology. 2007;19(4):408–15. 10.1016/j.coi.2007.06.004 . 17656078

[25] Hashimoto M, Kamphorst AO, Im SJ, Kissick HT, Pillai RN, Ramalingam SS, et al CD8 T Cell Exhaustion in Chronic Infection and Cancer: Opportunities for Interventions. Annual review of medicine. 2018;69:301–18. 10.1146/annurev-med-012017-043208 . 29414259

[26] Mueller SN, Matloubian M, Clemens DM, Sharpe AH, Freeman GJ, Gangappa S, et al Viral targeting of fibroblastic reticular cells contributes to immunosuppression and persistence during chronic infection. Proceedings of the National Academy of Sciences of the United States of America. 2007;104(39):15430–5. Epub 2007/09/20. 0702579104 [pii] 10.1073/pnas.0702579104 17878315 PMC2000533

[27] Scandella E, Bolinger B, Lattmann E, Miller S, Favre S, Littman DR, et al Restoration of lymphoid organ integrity through the interaction of lymphoid tissue-inducer cells with stroma of the T cell zone. Nature immunology. 2008;9(6):667–75. 10.1038/ni.1605 18425132

[28] Wei XQ, Charles IG, Smith A, Ure J, Feng GJ, Huang FP, et al Altered immune responses in mice lacking inducible nitric oxide synthase. Nature. 1995;375(6530):408–11. Epub 1995/06/01. 10.1038/375408a0 . 7539113

[29] Vig M, Srivastava S, Kandpal U, Sade H, Lewis V, Sarin A, et al Inducible nitric oxide synthase in T cells regulates T cell death and immune memory. The Journal of clinical investigation. 2004;113(12):1734–42. Epub 2004/06/17. 10.1172/JCI20225 15199408 PMC420501

[30] Bouffi C, Bony C, Jorgensen C, Noel D. Skin fibroblasts are potent suppressors of inflammation in experimental arthritis. Ann Rheum Dis. 2011;70(9):1671–6. Epub 2011/05/31. ard.2010.143297 [pii] 10.1136/ard.2010.143297 . 21623002

[31] Fletcher AL, Elman JS, Astarita J, Murray R, Saeidi N, D'Rozario J, et al Lymph node fibroblastic reticular cell transplants show robust therapeutic efficacy in high-mortality murine sepsis. Science translational medicine. 2014;6(249):249ra109 10.1126/scitranslmed.3009377 25122637 PMC4415170

[32] Yu M, Guo G, Zhang X, Li L, Yang W, Bollag R, et al Fibroblastic reticular cells of the lymphoid tissues modulate T cell activation threshold during homeostasis via hyperactive cyclooxygenase-2/prostaglandin E2 axis. Scientific reports. 2017;7(1):3350 10.1038/s41598-017-03459-5 28611431 PMC5469856

[33] Mueller SN, Vanguri VK, Ha SJ, West EE, Keir ME, Glickman JN, et al PD-L1 has distinct functions in hematopoietic and nonhematopoietic cells in regulating T cell responses during chronic infection in mice. The Journal of clinical investigation. 2010;120(7):2508–15. 10.1172/JCI40040 20551512 PMC2898584

[34] Uccelli A, Moretta L, Pistoia V. Mesenchymal stem cells in health and disease. Nat Rev Immunol. 2008;8(9):726–36. 10.1038/nri2395 . 19172693

[35] Knoblich K, Cruz Migoni S, Siew SM, Jinks E, Kaul B, Jeffery HC, et al The human lymph node microenvironment unilaterally regulates T-cell activation and differentiation. PLoS Biol. 2018;16(9):e2005046 10.1371/journal.pbio.2005046 . 30180168 PMC6122729

[36] Newberry RD, McDonough JS, Stenson WF, Lorenz RG. Spontaneous and continuous cyclooxygenase-2-dependent prostaglandin E2 production by stromal cells in the murine small intestine lamina propria: directing the tone of the intestinal immune response. Journal of immunology. 2001;166(7):4465–72. 10.4049/jimmunol.166.7.4465 . 11254702

[37] Broere F, du Pre MF, van Berkel LA, Garssen J, Schmidt-Weber CB, Lambrecht BN, et al Cyclooxygenase-2 in mucosal DC mediates induction of regulatory T cells in the intestine through suppression of IL-4. Mucosal immunology. 2009;2(3):254–64. 10.1038/mi.2009.2 . 19262503

[38] Kasic T, Colombo P, Soldani C, Wang CM, Miranda E, Roncalli M, et al Modulation of human T-cell functions by reactive nitrogen species. European journal of immunology. 2011;41(7):1843–9. 10.1002/eji.201040868 . 21480210

[39] Zhu Y, Zhu M, Lance P. iNOS signaling interacts with COX-2 pathway in colonic fibroblasts. Experimental cell research. 2012;318(16):2116–27. 10.1016/j.yexcr.2012.05.027 . 22683859

[40] Kim SF, Huri DA, Snyder SH. Inducible nitric oxide synthase binds, S-nitrosylates, and activates cyclooxygenase-2. Science. 2005;310(5756):1966–70. 10.1126/science.1119407 . 16373578

[41] Loftin CD, Tiano HF, Langenbach R. Phenotypes of the COX-deficient mice indicate physiological and pathophysiological roles for COX-1 and COX-2. Prostaglandins & other lipid mediators. 2002;68–69:177–85. . 12432917 10.1016/s0090-6980(02)00028-x

[42] Bottcher JP, Bonavita E, Chakravarty P, Blees H, Cabeza-Cabrerizo M, Sammicheli S, et al NK Cells Stimulate Recruitment of cDC1 into the Tumor Microenvironment Promoting Cancer Immune Control. Cell. 2018;172(5):1022–37 e14. 10.1016/j.cell.2018.01.004 29429633 PMC5847168

[43] Norberg JK, Sells E, Chang HH, Alla SR, Zhang S, Meuillet EJ. Targeting inflammation: multiple innovative ways to reduce prostaglandin E(2). Pharmaceutical patent analyst. 2013;2(2):265–88. 10.4155/ppa.12.90 24237030 PMC4028977

[44] Nakanishi M, Rosenberg DW. Multifaceted roles of PGE2 in inflammation and cancer. Seminars in immunopathology. 2013;35(2):123–37. 10.1007/s00281-012-0342-8 22996682 PMC3568185

[45] Chen DS, Mellman I. Oncology meets immunology: the cancer-immunity cycle. Immunity. 2013;39(1):1–10. 10.1016/j.immuni.2013.07.012 . 23890059

[46] Laubach VE, Shesely EG, Smithies O, Sherman PA. Mice lacking inducible nitric oxide synthase are not resistant to lipopolysaccharide-induced death. Proceedings of the National Academy of Sciences of the United States of America. 1995;92(23):10688–92. 10.1073/pnas.92.23.10688 7479866 PMC40677

[47] Hogquist KA, Jameson SC, Heath WR, Howard JL, Bevan MJ, Carbone FR. T cell receptor antagonist peptides induce positive selection. Cell. 1994;76(1):17–27. 10.1016/0092-8674(94)90169-4 . 8287475

[48] Schwenk F, Baron U., Rajewsky K. A cre-transgenic mouse strain for the ubiquitous deletion of loxP-flanked gene segments including deletion in germ cells. Nucleic Acids Res. 1995;23(24):5080–1. 10.1093/nar/23.24.5080 8559668 PMC307516

[49] Chai Q, Onder L, Scandella E, Gil-Cruz C, Perez-Shibayama C, Cupovic J, et al Maturation of lymph node fibroblastic reticular cells from myofibroblastic precursors is critical for antiviral immunity. Immunity. 2013;38(5):1013–24. 10.1016/j.immuni.2013.03.012 . 23623380 PMC7111182

[50] Srinivas S, Watanabe T, Lin CS, William CM, Tanabe Y, Jessell TM, et al Cre reporter strains produced by targeted insertion of EYFP and ECFP into the ROSA26 locus. BMC developmental biology. 2001;1:4 10.1186/1471-213X-1-4 11299042 PMC31338

[51] Battegay M, Cooper S, Althage A, Banziger J, Hengartner H, Zinkernagel RM. Quantification of lymphocytic choriomeningitis virus with an immunological focus assay in 24- or 96-well plates. Journal of virological methods. 1991;33(1–2):191–8. . 1939506 10.1016/0166-0934(91)90018-u

