# Supplementary figures and images for: Attenuation of chronic antiviral T-cell responses through constitutive COX2-dependent prostanoid synthesis by lymph node fibroblasts
Source: PLoS Biol. 2019 Jul 15;17(7):e3000072. doi: 10.1371/journal.pbio.3000072 (PMC6657915; doi:10.1371/journal.pbio.3000072)

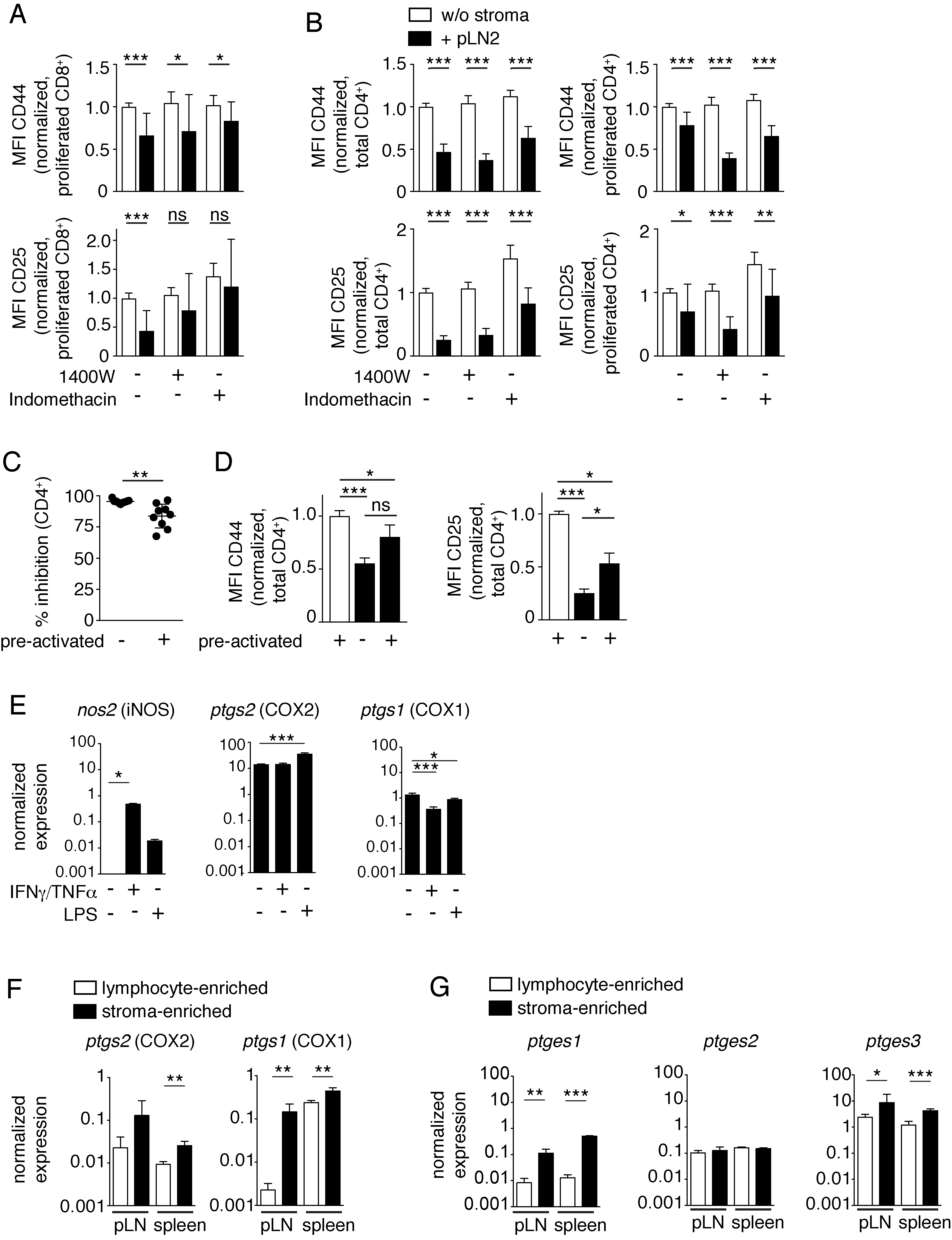

Supplement: S1 Fig — (A–B) T cells were activated with αCD3/28 DynaBeads and cultured for 3 d in the absence (white bars) or presence of pLN2 (black bars) ± 1400W (3 μM) ± indomethacin (10 μM). Flow cytometric analysis showing the MFI of CD44 and CD25 on proliferated CD8+ T cells (A) and total or proliferated CD4+ T cells (B). Data pooled from four independent experiments, n = 10. (C, D) CFSE-labeled T cells were either activated with αCD3/28 DynaBeads and cultured ± pLN2 or preactivated for 24 h with αCD3/28 DynaBeads followed by 2 d culture + pLN2. (C) Scatter plot showing percentage inhibition of CD4+ T cells mediated by pLN2. (D) MFI of CD44 and CD25 of CD4+ T cells cocultured with pLN2 and normalized to the MFI of CD4+ T cells cultured without pLN2. Data shown in (C) and (D) represent a pool of 3 independent experiments (n = 9). (E) RT-qPCR analysis for Nos2, ptgs2, and ptgs1 transcript levels in pLN2 cells that were left unstimulated or stimulated for 7 h with 10 ng/ml of both IFNγ and TNFα or with 0.5 μg/ml LPS (n = 3). (F–G) RT-qPCR analysis of the soluble (lymphocyte-enriched) and nonsoluble (stroma-enriched) fractions of pLNs and spleens of naïve WT mice (n = 4) for transcripts of ptgs2/1 (F) or ptges1/2/3 (G). All bar graphs indicate the mean ± SD. Statistics: (A), (B), (C), (F), and (G) using unpaired t test or Mann–Whitney, respectively. (D and E) ANOVA or Kruskal–Wallis, followed by multiple comparisons test. *P < 0.05, **P < 0.005, and ***P < 0.001. Data used in the generation of this figure can be found in S1 Data. CD, cluster of differentiation; CFSE, carboxyfluorescein succinimidyl ester; COX, cyclooxygenase; d, day; IFN, interferon; iNOS, inducible nitric oxide synthase; LN, lymph node; LPS, lipopolysaccharide; MFI, median fluorescence intensity; pLN, peripheral LN; ptgs, prostaglandin-endoperoxide synthase; RT-qPCR, reverse transcription followed by a quantitative polymerase chain reaction; SD, standard deviation; TNF, tumor necrosis factor; WT, wild type. (TIF) [file pbio.3000072.s002.tif]

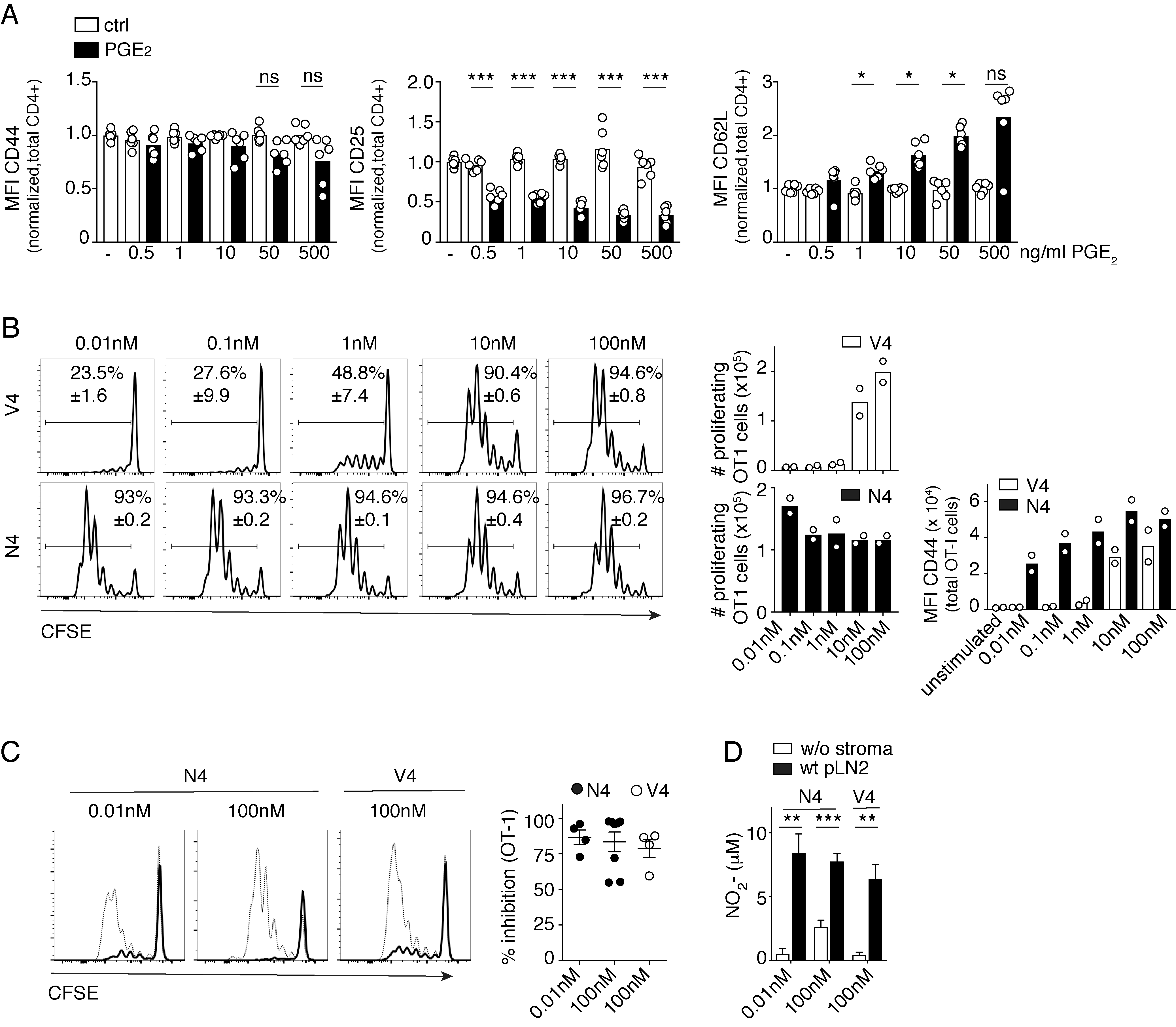

Supplement: S2 Fig — Flow cytometric analysis of CFSE-labeled T cells activated in vitro for 3 d, either polyclonally with αCD3/28-coated beads (A) or with peptide antigens presented by BMDCs (B–C). (A) T cells cultured in absence of pLN2 but with the indicated concentrations of PGE2 (black bars) or solvent control (white bars), respectively. Shown is the MFI of CD44, CD25, and CD62L on total CD4+ T cells, normalized to the MFI of untreated cells in order to see the fold difference in the expression level upon treatment. n = 6; pool of 3 independent experiments. (B) CFSE-labeled OT-1 CD8+ T cells were mixed in a ratio of 1:50 with WT T cells and cultured with LPS-activated BMDCs pulsed with the indicated concentrations of OVA peptides of high affinity (N4) or low affinity (V4) for the OT-1 receptor, ± pLN2 FRCs. OT-1 cell proliferation or activation (B, C) or nitrite levels (D) were assessed after 3 d of culture. (B) CFSE profiles (left side), numbers (middle panel), and CD44 expression levels (right panel) of OT-1 T cells activated in the absence of the pLN2 FRC line. Data are representative of 2 independent experiments performed in duplicates. (C) CFSE profile of OT-1 T cells cultured in the absence (thin line) or presence (black line) of pLN2 FRCs. Scatter dot plot depicts the percentage inhibition of OT-1 T-cell proliferation by FRCs. (D) Bar graphs showing nitrite (NO2−) levels found in the supernatant of the cocultures shown in (C). Data in (C) and (D) represent a pool of 2 independent experiments; n ≥ 4. Statistics: (A and D) unpaired t test or Mann–Whitney test was performed. *P < 0.05, **P < 0.005, and ***P < 0.001. Data used in the generation of this figure can be found in S1 Data. BMDC, bone-marrow–derived dendritic cell; CD, cluster of differentiation; CFSE, carboxyfluorescein succinimidyl ester; d, day; FRC, fibroblastic reticular cell; LN, lymph node; LPS, lipopolysaccharide; MFI, median fluorescence intensity; OT-1, ovalbumin-specific CD8+ T cell; PGE2, prostaglandin E2; [file pbio.3000072.s003.tif]

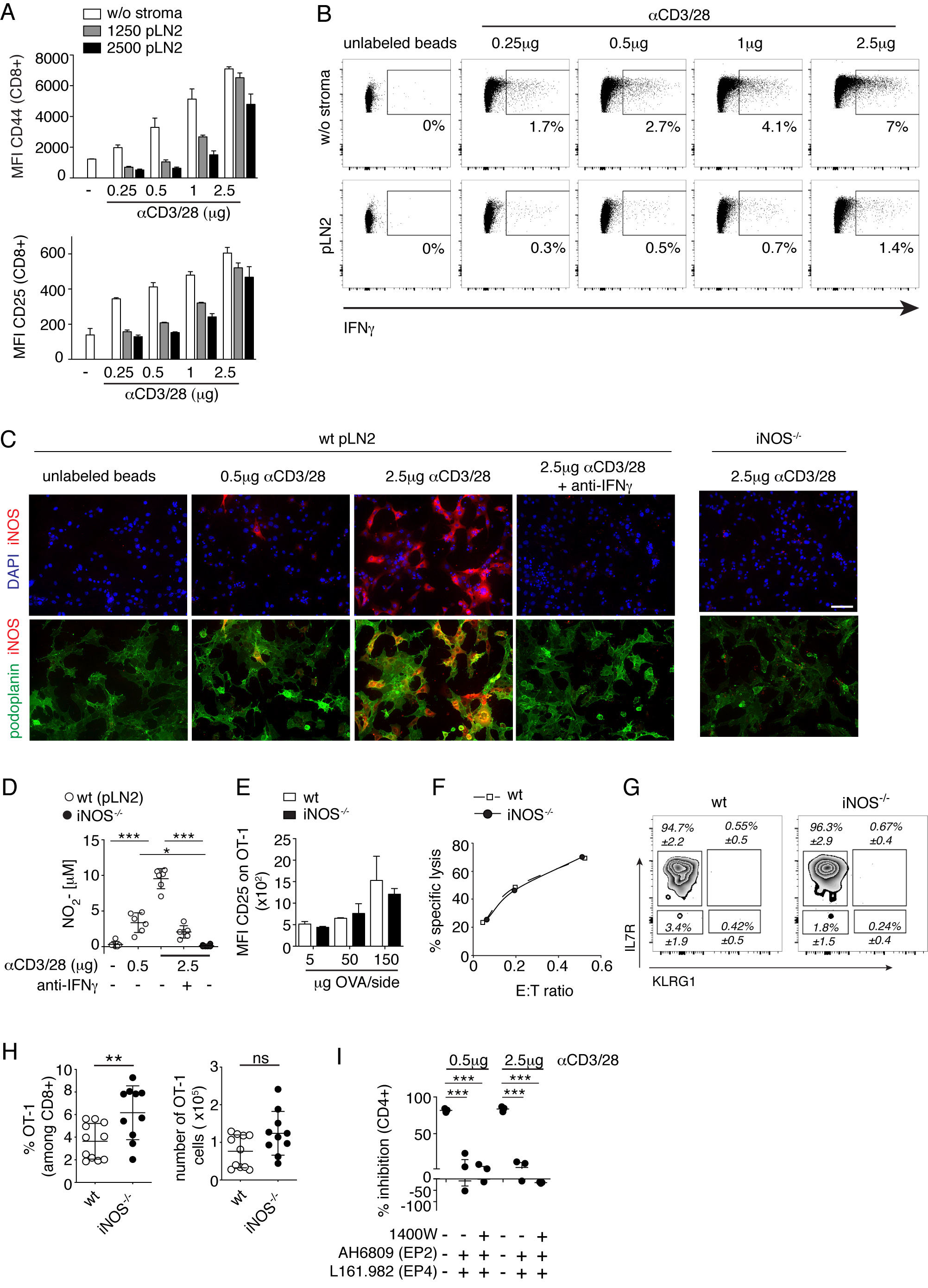

Supplement: S3 Fig — (A–D) CD8+ and CD4+ T cells were activated with the indicated amount of αCD3/28-coated onto MicroBeads ± pLN2 FRCs. (A) MFI of CD8+ T cells cultured for 3 d ± pLN2 in the indicated numbers. Data are representative of 4–5 independent experiments with 3 replicates each. (B) The frequency of IFNγ-producing CD8+ T cells were investigated after 1 d of coculture. One representative out of 2–3 independent experiments is shown, with at least 2 replicates in each experiment. (C) Histological analysis of d 2 cocultures containing FRCs and activated CD8+ and CD4+ T cells for iNOS protein expression in pdpn+ FRCs. DAPI highlights cell nuclei. Some cultures contained neutralizing anti-IFNγ antibodies. Scale bar, 100 μm. Shown photos are representative of 3 independent experiments. (D) WT (pLN2) and iNOS−/− FRC cell lines were cocultured with activated T cells ± anti-IFNγ antibodies, and nitrite levels measured in the SN of d 2 cultures using the Griess assay. Scatter plot showing 1 representative out of 3 independent experiments. (E–G) WT or iNOS−/− mice that had received OT-1 CD8+ T cells IV were immunized SC with OVA/Montanide and the draining pLNs investigated on d 4 after immunization. (E) Bar graphs depict the MFI of CD25 expression on OT-1 T cells isolated from WT versus iNOS−/− mice immunized with the indicated concentrations of OVA (n ≥ 8, pool of 2–3 independent experiments). (F) Cytotoxic capacity of OT-1 T cells isolated from draining pLNs of WT and iNOS−/− mice immunized SC 4 d earlier with 150 μg OVA/Montanide/Poly(I:C). Shown is the percentage of target cell lysis with the indicated E:T ratios for one representative (n = 3) out of 2 independent experiments. (G) Memory phenotype of CD44+ OT-1 T cells (CD45.1 + CD8α+) in draining pLNs on d 40 after SC immunization with 50 μg OVA/Montanide/Poly(I:C). n = 7. (H) The indicated mice received a primary immunization SC in the left flank with 50 μg OVA in Montanide/Poly(I:C) adjuvant and were challenged on d 37 after primar [file pbio.3000072.s004.tif]

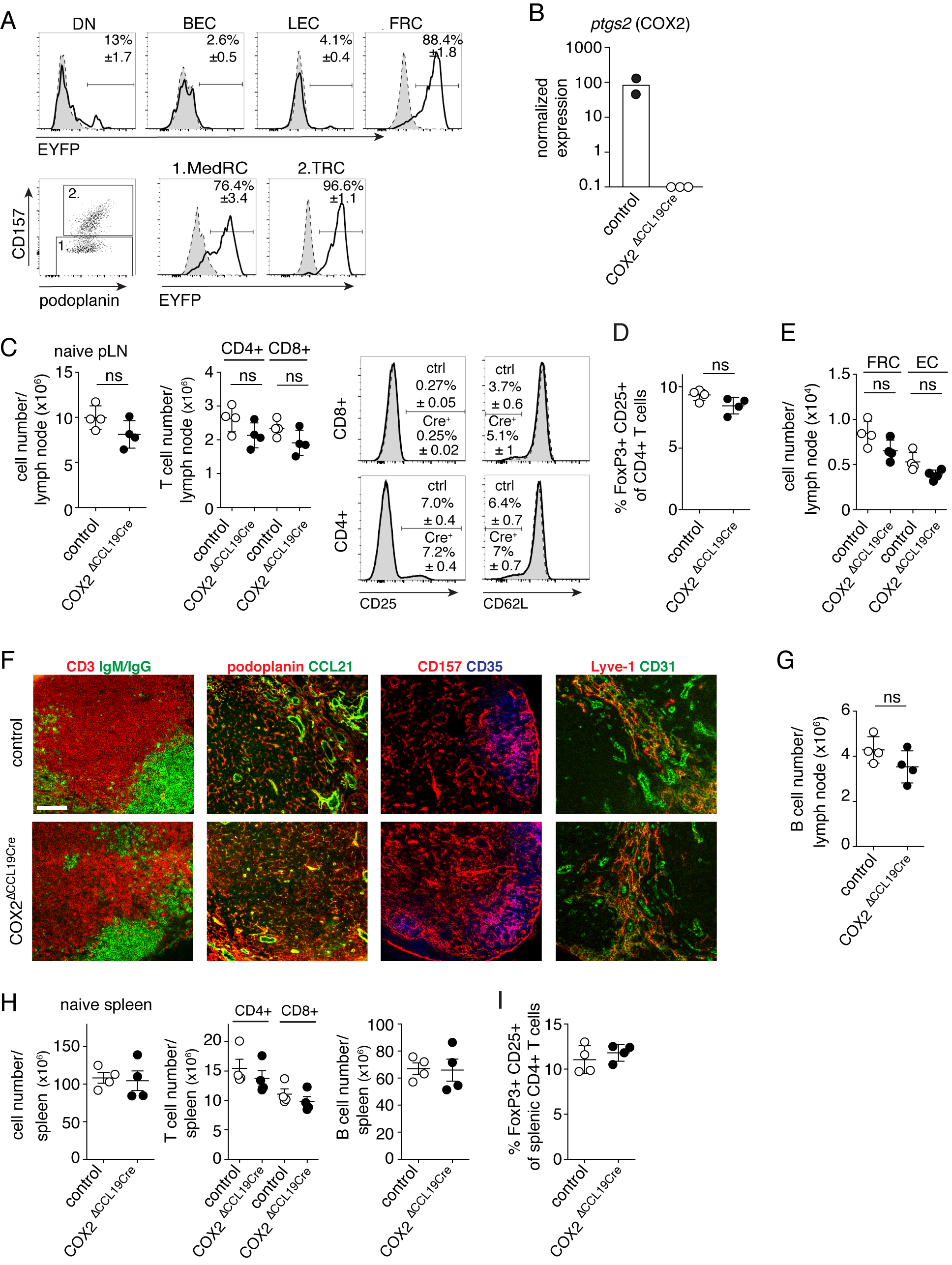

Supplement: S4 Fig — Characterization of pLNs and spleens of naïve mice genetically lacking COX2 expression specifically in FRCs (COX2ΔCCL19Cre) versus their littermate Cre− mice (called “controls”). (A) CCL19Cre activity was investigated in pLNs of naïve COX2ΔCCL19Cre ROSA26-EYFPCCL19Cre reporter mice. Histograms showing EYFP expression in different nonhematopoietic cell types (CD45−): CD31− pdpn− DN cells, CD31+ pdpn− BECs, CD31+ pdpn+ LECs, and CD31− pdpn+ FRCs in EYFP reporter (solid black line) compared to control Cre− mice (gray shading). FRCs were further divided into CD157− MedRCs and CD157+ TRCs. n = 3, representative data of 2 independent experiments. (B–G) Characterization of naïve pLNs of COX2ΔCCL19Cre mice. (B) Transcript levels of ptgs2 were analyzed in sorted TRCs (CD45− CD35− CD31− pdpn+ CD157+). n = 2–3; each sample represents a pool of 2–3 mice. Transcript levels below the detection limit or nonspecific transcripts are indicated as white circles on the x-axis. (C) Scatter plots showing total cell numbers (left side) or CD4+ and CD8+ T-cell numbers (middle) in digested naïve pLNs or representative histograms (right side) showing the percentage of activated CD8+ and CD4+ T cells by gating on CD25high or CD62Llow cells in samples derived from Cre+ (thick line) or Cre− littermates (dashed line with gray shading). (D, E) Scatter plots showing percentage of FoxP3+ CD25+ Treg among CD4+ T cells (D) and total FRC and EC numbers (E). (F) Immunofluorescence microscopy analysis of labeled pLN sections. Localization of T and B cells, as well as antibody staining for different stromal cell types or their products, is shown. Data are representative for 2 independent experiments investigating 3 mice per genotype. Scale bar, 100 μm. (G) Scatter plots showing CD19+ B-cell numbers in pLN. (H–I) Flow cytometric characterization of naïve spleens of COX2ΔCCL19Cre mice. Shown are scatter plots depicting total cell numbers, lymphocyte numbers (H), and Treg cell numbers (I). Data in (C), (D), [file pbio.3000072.s005.tif]

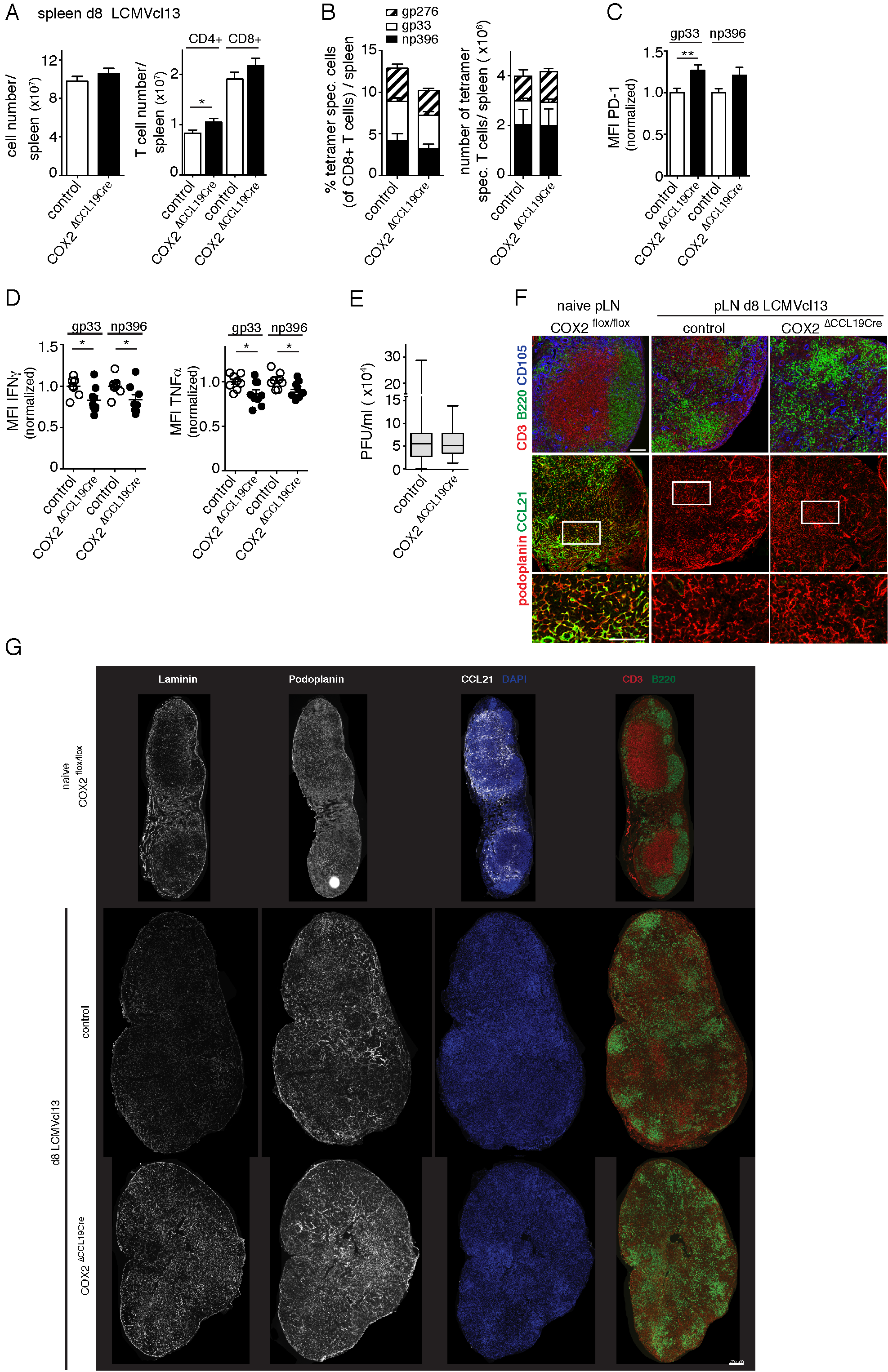

Supplement: S5 Fig — COX2ΔCCL19Cre and Cre− littermate mice were infected with 2 × 10^6^ PFU LCMV clone 13 and the spleens and pLNs analyzed on d 8 p.i.. Bar graphs showing (A) total cell numbers and T-cell numbers as well as (B) frequencies and cell numbers of LCMV-specific CD8+ T cells (specific for three viral peptides) in the spleen. Shown is a pool of 2 independent experiments; n = 9, except for gp276 tetramer+ cells with n = 4 of 1 experiment. (C) MFI of PD-1 on splenic LCMV-specific CD8+ T cells, with cells from Cre+ mice normalized to those from Cre− mice. (D) Frequencies of splenic IFNγ- and TNFα-expressing LCMV-specific T cells determined after short restimulation with gp33 or np396 peptides, respectively. Scatter plot showing normalized frequencies of LCMV-specific cells of Cre+ compared to Cre− controls. Data in (C) and (D) show a pool of 2 independent experiments; n ≥ 5. (E) Box and whisker plots showing viral titer in the blood (n ≥ 7). (F) Immunofluorescence microscopy analysis of labeled pLN sections from naïve or d 8 LCMV-clone-13–infected mice of the indicated genotype. Representative images from 3 mice/genotype are depicted. Scale bar, 100 μm. (G) Stitched images of labeled pLN sections described in (F). Scale bar, 200 μm. Bar graphs and scatter plots showing mean ± SEM. Statistics: unpaired t test or Mann–Whitney test. *P < 0.05, **P < 0.005, and ***P < 0.001. Data used in the generation of this figure can be found in S1 Data. CCL19cre, CCL19 promoter driving Cre recombinase expression; CD, cluster of differentiation; COX, cyclooxygenase; d, day; FRC, fibroblastic reticular cell; gp33, LCMV glycoprotein 33–41 peptide; IFN, interferon; LCMV, lymphocytic choriomeningitis virus; LN, lymph node; MFI, median fluorescence intensity; np396, LCMV nucleoprotein 396–404 peptide; PFU, plaque-forming unit; pLN, peripheral LN; p.i., postinfection; TNF, tumor necrosis factor. (TIF) [file pbio.3000072.s006.tif]

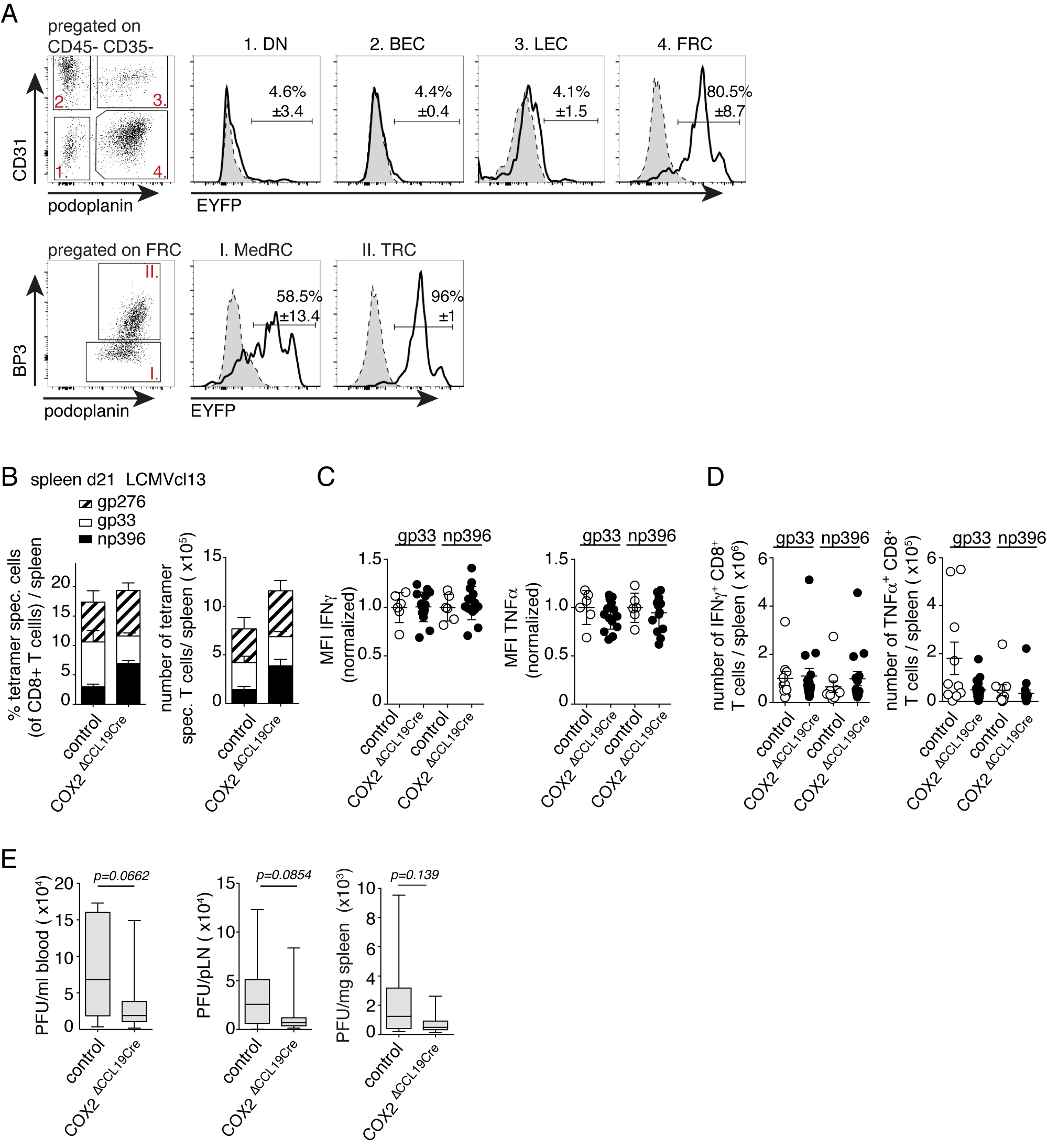

Supplement: S6 Fig — COX2ΔCCL19Cre mice were infected with LCMV clone 13 and pLNs and spleens analyzed during the chronic phase at d 19–21 after infection, using flow cytometry (A–D) or plaque-forming assay (E). (A) pLN of COX2ΔCCL19Cre ROSA26-EYFPCCL19Cre mice were digested enzymatically and analyzed on d 19 p.i.. Cre activity was investigated by measuring EYFP levels in different cell types using flow cytometry. Dot plot shows gating strategy to distinguish CD31− pdpn− DN cells, CD31+ pdpn− BECs, CD31+ pdpn+ LECs, and CD31− pdpn+ FRCs after pregating on CD45− CD35− nonhematopoietic cells. The FRC population was further subdivided into CD157− MedRCs and CD157+ TRCs. The histograms show the frequency of EYFP-expressing cells among different stromal cell subsets, with the cells from Cre− mice shown with gray shading and those from Cre+ mice in black lines. One out of two representative experiments is shown (n = 3). (B–D) The spleens of COX2ΔCCL19Cre and control mice were analyzed on d 21 post-clone 13 infection for the frequency and number of LCMV-specific CD8+ T cells (B), for the MFI of intracellular IFNγ or TNFα levels in virus-specific CD8+ T cells (C), or for the number of IFNγ- or TNFα-expressing splenic CD8+ T cells (D; normalized to controls) after restimulation with gp33 or np396 peptides, respectively. (C, D) Pool of 3 independent experiments; n ≥ 6. (B–D) Bar graphs and scatter plots showing mean ± SEM. (E) Box and whisker plots showing viral titers in the blood, pLNs, and spleens of d 21 LCMV-clone-13–infected Cre− and Cre+ mice. Statistics: unpaired t test or Mann–Whitney test. *P < 0.05, **P < 0.005, and ***P < 0.001. Data used in the generation of this figure can be found in S1 Data. BEC, blood endothelial cell; CCL19cre, CCL19 promoter driving Cre recombinase expression; CD, cluster of differentiation; COX, cyclooxygenase; d, day; DN, double negative; EYFP, enhanced yellow fluorescent protein; FRC, fibroblastic reticular cell; IFN, interferon; LCMV, lymphocytic choriomeni [file pbio.3000072.s007.tif]
